# Comprehensive analysis of PLOD family members in low-grade gliomas using bioinformatics methods

**DOI:** 10.1371/journal.pone.0246097

**Published:** 2021-01-27

**Authors:** Yonghui Zhao, Xiang Zhang, Junchao Yao

**Affiliations:** Department of Neurosurgery, Cangzhou Central Hospital, Cangzhou, Hebei, People’s Republic of China; University of Calgary, CANADA

## Abstract

Low-grade gliomas (LGGs) is a primary invasive brain tumor that grows slowly but is incurable and eventually develops into high malignant glioma. Novel biomarkers for the tumorigenesis and lifetime of LGG are critically demanded to be investigated. In this study, the expression levels of procollagen-lysine, 2-oxoglutarate 5-dioxygenases (PLODs) were analyzed by ONCOMINE, HPA and GEPIA. The GEPIA online platform was applied to evaluate the interrelation between PLODs and survival index in LGG. Furthermore, functions of PLODs and co-expression genes were inspected by the DAVID. Moreover, we used TIMER, cBioportal, GeneMINIA and NetworkAnalyst analysis to reveal the mechanism of PLODs in LGG. We found that expression levels of each PLOD family members were up-regulated in patients with LGG. Higher expression of PLODs was closely related to shorter disease-free survival (DFS) and overall survival (OS). The findings showed that LGG cases with or without alterations were significantly correlated with the OS and DFS. The mechanism of PLODs in LGG may be involved in response to hypoxia, oxidoreductase activity, Lysine degradation and immune cell infiltration. In general, this research has investigated the values of PLODs in LGG, which could serve as biomarkers for diagnosis, prognosis and potential therapeutic targets of LGG patients.

## Introduction

LGGs are common tumors in the central nervous system, which can progress into high-grade glioma, leading to undesirable prognosis [1, 2]. Advances in genome sequencing have elucidated the genetic and novel biomarkers of high-grade glioma, which provided newly categorization and some promising treatments [3, 4]. However, the molecular mechanisms and targeted gene markers for LGGs are poorly understood, so more promising and therapeutic biomarkers are urgently needed.

PLOD family is composed of three members, PLOD 1/2/3, which is a group of enzymes engaged in stabilizing collagen through cross-linking and hydroxylation of lysine [5, 6]. PLOD family members are the lysyl hydroxylase responsible for the lysyl hydroxylation of collagen [7, 8]. Molecular biology mechanisms of PLOD family involving a wide range of biological processes, such as modulating cancer cell migration, tumorigenesis and development [9]. Many studies show that over expression of PLODs can promote tumor invasion and higher recurrence, suggesting that targeting PLOD family members is potential strategy for cancer treatment [10]. However, the effect of this promising gene family in LGGs is still lacking research.

In the current study, we studied the expression levels and prognosis of PLODs in LGGs based on online databases, platforms, and various data sets. The purpose of this study is to provide insights into the molecular mechanism of LGG and uncover potential new biomarkers for the disease.

## Materials and methods

### ONCOMINE analysis

According to the **ONCOMINE** (https://www.oncomine.org/) dataset [11], we tested the transcription levels of PLODs in different tumors. Besides, we compared the expression of PLODs between the subtypes in LGG and normal tissues, ‘PLOD1, PLOD2 and PLOD3’ was selected as the keywords in search, and ‘Anaplastic Astrocytoma vs. Normal Analysis’ was chosen as Analysis Type. The threshold was set up as *P*-value 0.05, fold change 2, and top 10% gene rank.

### HPA analysis

HPA dataset (https://www.proteinatlas.org/) is an open access to enable researchers to freely access data for exploration of the human protein in different tissues [12, 13]. The HPA database was also used to validate the immunohistochemistry of the PLODs in patients with LGG. According to the fraction of stained cells, staining quantity was also divided into four levels: none, <25%, 25–75%, and >75%. Protein expression levels were based on staining intensity and staining quantity. The classification criteria for protein expression levels were as follows: negative, not detected; weak and <25%, not detected; weak combined with either 25–75% or 75%, low; moderate and <25%, low; moderate combined with either 25–75% or 75%, medium; strong and <25%, medium; and strong combined with either 25–75% or 75%, high.

### GEPIA analysis

The GEPIA database (http://gepia.cancer-pku.cn/) is an online dataset for comparing the gene expression profile in cancer and paired normal tissues [14]. The prognostic values of high and low expression PLODs in LGG were analyzed using GEPIA. The PLODs expression threshold of 50% (median value) was determined to split the PLOD1/2/3 high-expression and low-expression cohorts. Therefore, samples with PLOD family gene expression levels higher and lower than 50% were divided as the high-expression sample and the low-expression sample, separately. Overall survival and the disease-free survival analysis were also conducted on the basis of PLOD family gene expression. Regarding hypothesis test, the GEPIA considers the Log-rank test. For this, we selected a hazards ratio (HR) based on the Cox PH model.

### cBioPortal analysis

The cBio Cancer Genomics Portal(cBioPortal) (http://cbioportal.org) was applied to investigated the genetic mutations of PLODs in LGG [15]. Mutation and a summary of the gene types in LGG was inquiry. According to cBioPortal’s online instructions, DFS and OS were analyzed for with or without PLODs mutation in LGG.

### GeneMINIA and NetworkAnalyst analysis

GeneMANIA (http://www.genemania.org) [16] was used to analyze the relationship between the PLOD family members and co-expression genes. Using the NetworkAnalyst (https://www.network analyst.ca/) [17], we analyzed 23 correlated genes screening by GeneMINIA database, which integrates pathway, genetic interactions, co-expression genes and physical interactions.

### DAVID

To reveal the functions of PLODs and the twenty interactors from GeneMINIA analysis, DAVID database (https://david.ncifcrf.gov/) was used to explore the functions of PLODs in LGG [18]. In this research, Gene Ontology (GO) and Kyoto Encyclopedia of Genes and Genomes (KEGG) pathway enrichment analyses of PLOD family members and their 20 closely related neighbor genes were conducted using DAVID tool. GO was the biological process from the molecular to the organism level network construction and module analysis, including molecular function (MF), biological processes (BP) and cellular components (CC). The cutoff value for significant GO terms and KEGG pathways was a false discover rate (FDR) of <0.05. The enriched GO terms and pathways of genes were ranked by enrichment score (−log10 (P value)).

### TIMER analysis

TIMER is an internet platform resources for comprehensive investigation of the relationship between immune cells and multiple cancer types (https://cistrome.shinyapps.io/timer/) [19]. TIMER applies algorithm method to evaluate the abundance of tumor-infiltrating immune cells from gene expression profiles. In this dataset, we analyzed the correlation of PLODs expression with the abundance of immune infiltrates in LGG.

## Results

### Transcriptional levels of PLODs in LGG

**Fig 1:**
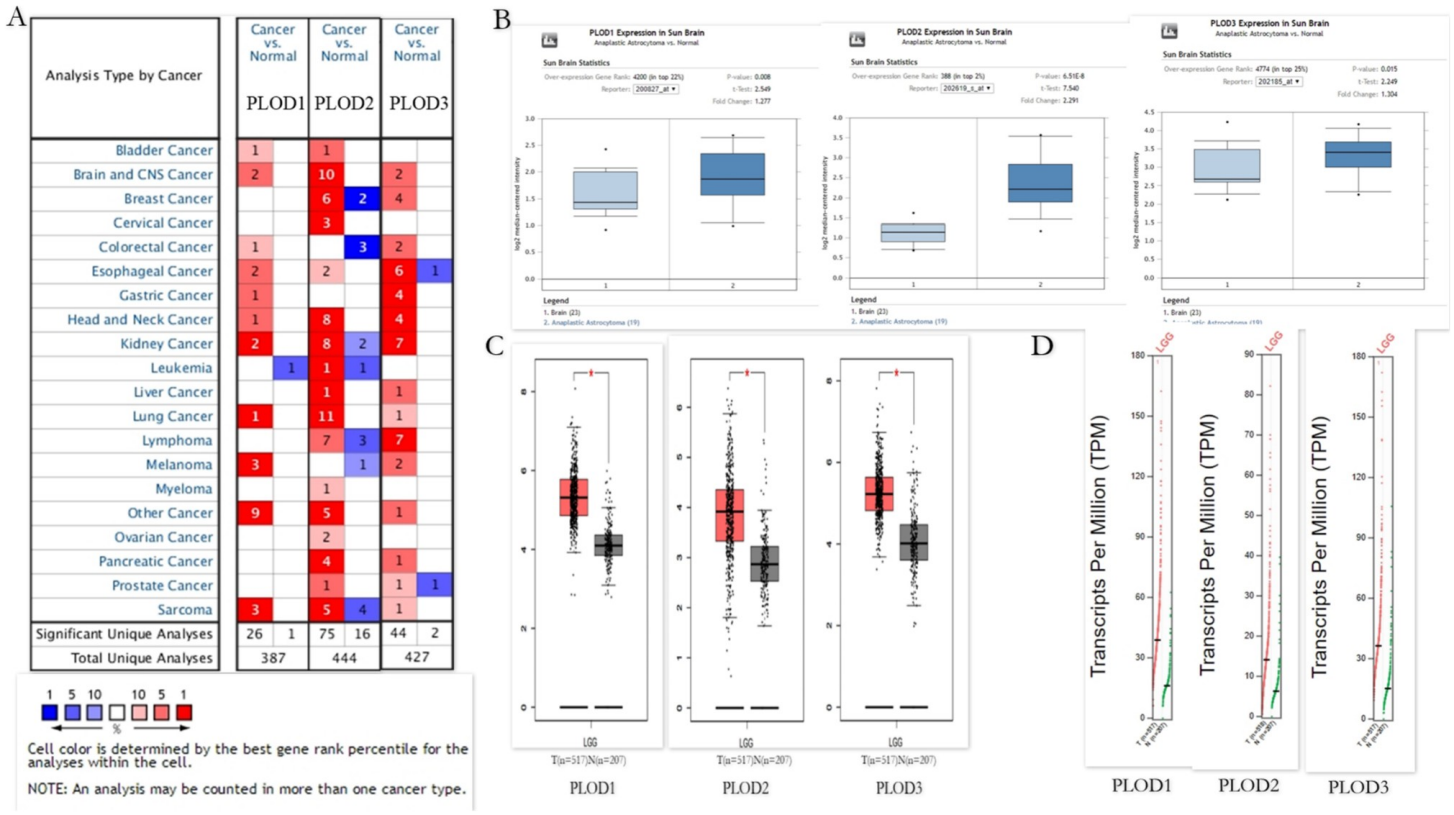
The expression of PLOD family members in different tissues. (A) The transcription levels of PLOD family members in different types of cancers Red, upregulation; blue, downregulation. The number in each cell indicates the datasets that met the set threshold in each cancer type. Cell color was defined as the gene rank percentile for analyses within the cell. (B) Expression levels of PLODs family members compared between subtypes of LGG and normal tissues from the Oncomine. (C) Boxplot results of the expression levels of PLODs family members in LGG analyzed using GEPIA. Red box, tumor samples; green box, normal samples. T, tumor; N, normal. (D) Expression profile of PLODs family members in LGG analyzed using GEPIA. Red trace, tumor samples; green trace, normal samples.

Diverse transcriptional levels of PLODs have been investigated in twenty human cancers and adjacent normal tissues in the **Oncomine**. As illustrated in Fig 1A, we have compared the transcriptional levels of PLODs in cancers with those in the normal tissues. In contrast to normal specimens, PLODs mRNA levels of all members were over expression in Brain and CNS Cancers. PLOD1 overexpression was illustrated in 2 datasets [20], followed by PLOD2 in 10 datasets [20–26], and PLOD3 in 2 datasets. All the datasets were summarized in Table 1. Using the **Oncomine** database, we compared the mRNA expression of PLODs in the subtypes of LGG with normal brain tissues. The Fig 1B showed that the expression levels of PLODs were all observed significantly higher in anaplastic astrocytoma as compared with the normal tissues. We also used the GEPIA database to compare the expression of PLODs between LGG and normal brain tissues. Contrast to normal brain tissues, the expression level of PLODs in LGG was significantly up-regulated (Fig 1C and 1D).

**Table 1:** The upregulated mRNA expressions of PLOD family genes in GC from ONCOMINE database*.

PLOD family genes	Fold change	*P*- Value	Dataset	Group comparison
PLOD1				
	3.742	1.33E-10	TCGA Brain	Brain Glioblastoma vs. Normal
	2.082	2.96E-5	Bredel Brain 2	Glioblastoma vs. Normal
PLOD2				
	3.543	1.71E-25	Sun Brain	Glioblastoma vs. Normal
	2.356	0.004	Sun Brain	Diffuse Astrocytoma vs. Normal
	3.494	3.40E-11	Bredel Brain 2	Glioblastoma vs. Normal
	2.688	1.45E-4	Rickman Brain	Astrocytoma vs. Normal
	3.204	6.26E-7	Shai Brain	Glioblastoma vs. Normal
	6.368	2.75E-12	TCGA Brain	Brain Glioblastoma vs. Normal
	3.326	0.014	Liang Brain	Glioblastoma vs. Normal
	2.430	0.002	French Brain	Anaplastic Oligoastrocytoma vs. Normal
	2.716	0.004	Lee Brain	Glioblastoma vs. Normal
PLOD3				
	2.873	1.03E-10	TCGA Brain	Brain Glioblastoma vs. Normal
	2.204	0.003	TCGA Brain	Glioblastoma vs. Normal

*Only datasets that meet the criteria *P* value < 0.05 and fold change > 2 are listed.

In summary, the results showed that the transcriptional levels of PLODs were up-regulated in multiple online datasets.

### Protein expression levels of PLODs in the human protein atlas

In order to further investigate the expression of PLODs at the protein level, we further verified their expression levels using the Human Protein Atlas (**HPA**) database. The direct link to these results in the HPA are as follows: https://www.proteinatlas.org/ENSG00000083444-PLOD1/tissue/cerebral+cortex#img (PLOD1, in normal tissues); https://www.proteinatlas.org/ENSG00000083444-PLOD1/pathology/glioma#img (PLOD1, in tumor tissues); https://www.proteinatlas.org/ENSG00000152952-PLOD2/tissue/cerebral+cortex#img (PLOD2, in normal tissues); https://www.proteinatlas.org/ENSG00000152952-PLOD2/pathology/glioma#img (PLOD2, in tumor tissues); https://www.proteinatlas.org/ENSG00000106397-PLOD3/tissue/cerebral+cortex#img (PLOD3, in normal tissues); https://www.proteinatlas.org/ENSG00000106397-PLOD3/pathology/glioma#img (PLOD3, in tumor tissues).

In summary, the present results indicated that PLOD1 was expressed at low staining in LGG tissues and was not detected in normal tissues. Immunohistochemistry for the PLOD2 in the HPA database showed that PLOD2 were low and medium staining in LGG tissues. In another, PLOD3 was not detected between LGG and normal tissues.

### Prognostic values of PLODs in patients with LGG

**Fig 2:**
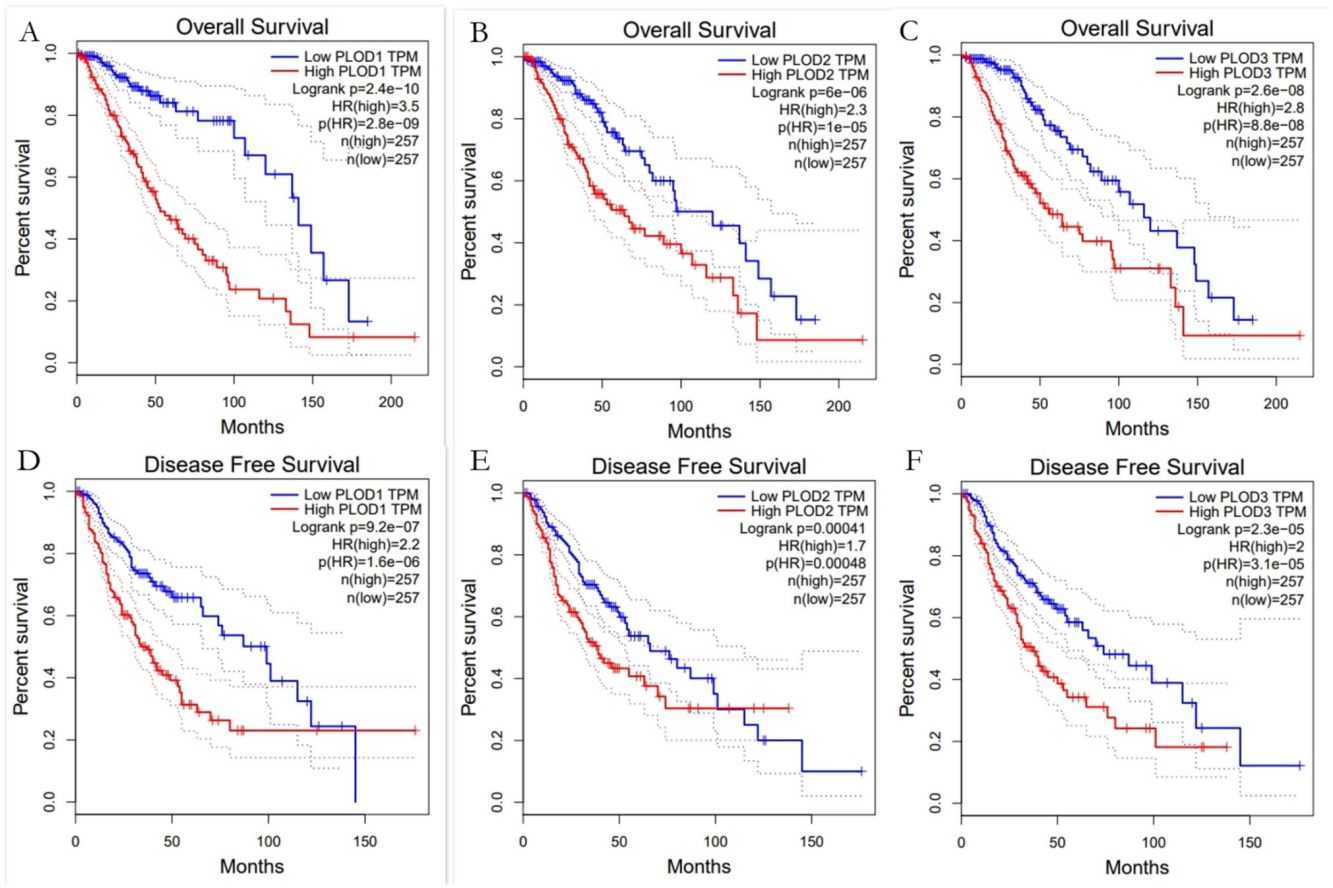
The survival analysis of PLOD family members in LGG. (A-C) Overexpression level of PLOD1, PLOD2 and PLOD3 were associated with shorter OS in LGG. (D-F) Overexpression level of PLOD1, PLOD2 and PLOD3 were associated with shorter DFS in LGG.

To explore the prognostic value of PLODs in patients with LGG, **GEPIA** analysis was performed according to the mRNA expression of individual PLODs family members. The findings showed that the mRNA expression levels of PLODs were closely associated with shorter OS and DFS in patients with LGG (Fig 2). The present results suggested that patients with LGG with a high mRNA expression level of PLOD1/2/3 were predicted lower OS and DFS.

In brief, PLOD family members may be biomarkers for poor prognosis in patients in LGG.

### Genetic alteration analysis of PLODs in LGG

**Fig 3:**
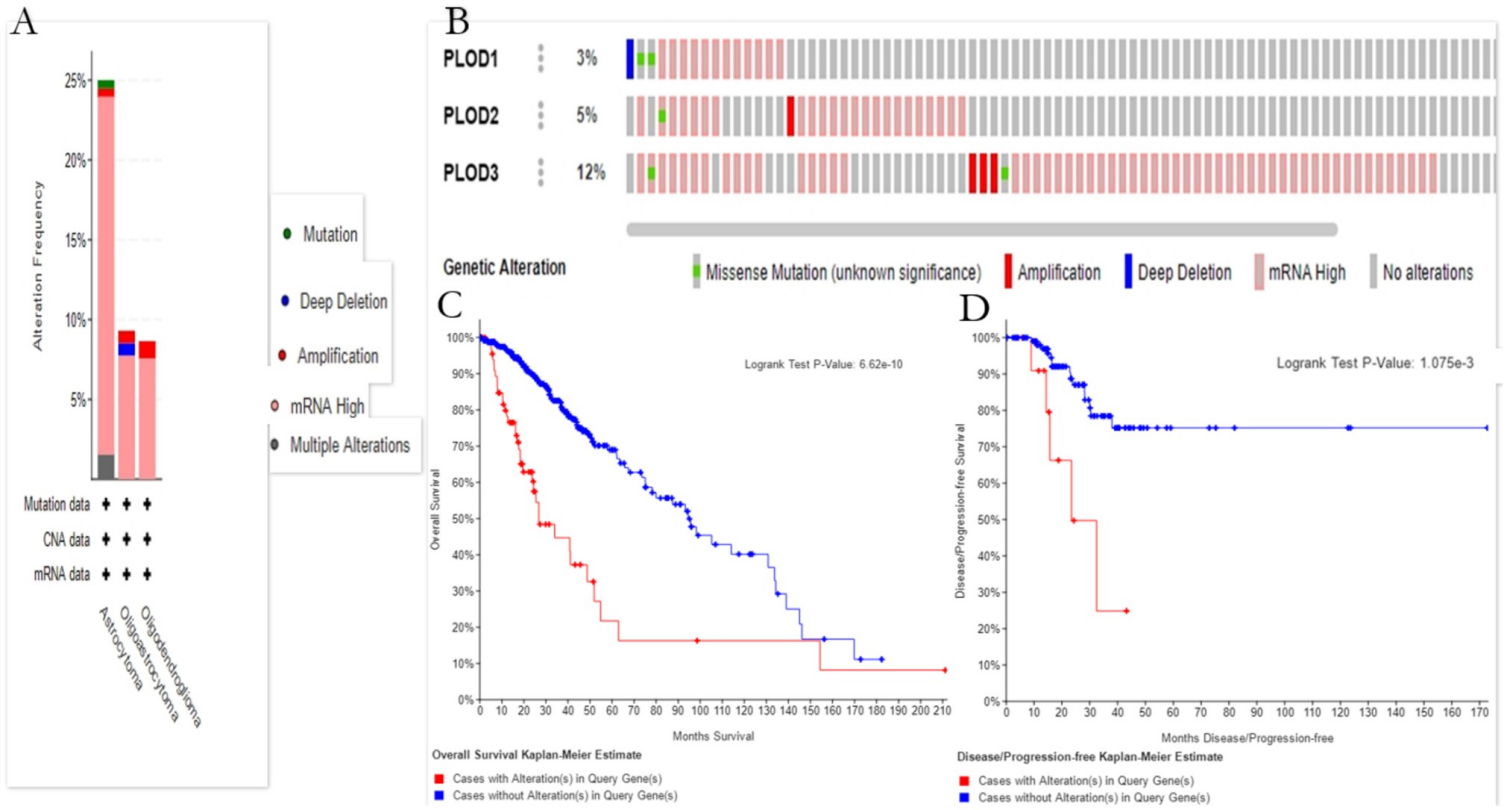
Alteration frequency and prognosis of the PLOD family genes in patients with LGG. (A) The summary of the cancer types in the cBioPortal was used to calculate the percentages of LGG cases with of the PLOD family genes. (B) mRNA expression alterations (RNA Seq V2 RSEM) of the PLOD family genes in LGG patients. (C) OS and DFS/PFS of LGG patients with altered (red) and unaltered (blue) mRNA expression of the PLOD family genes. The red curves in the Kaplan-Meier plots includes all tumors with PLOD1/2/3 germline or somatic mutation, the blue curves include all samples without PLOD1/2/3 mutation.

In another, the **cBioPortal** online tool was used to analyze the genetic variation of PLOD family members in LGG patients. The results indicated that three categories are shown based on filtering. (Fig 3A). The ratios of genetic alterations in PLOD family members for LGG different from 3% to 12% for each member (PLOD1 3%; PLOD2 5%; PLOD3 15%) (Fig 3B). Furthermore, we analyzed genetic alteration in PLODs and their associations with the OS and DFS of LGG patients. As was shown in Fig 3C and 3D, the results indicated that LGG cases with or without alterations were significantly related with the OS and DFS.

The genetic alteration analysis showed a significantly shorter overall and disease-free survival of patients with PLODs mutation in LGG patients. We speculate that detecting mutations in the PLOD gene family members will help determine the prognosis of LGG patients.

### Construction of interactive genes network and TF-miRNA coregulation network of PLODs family members in LGG

**Fig 4:**
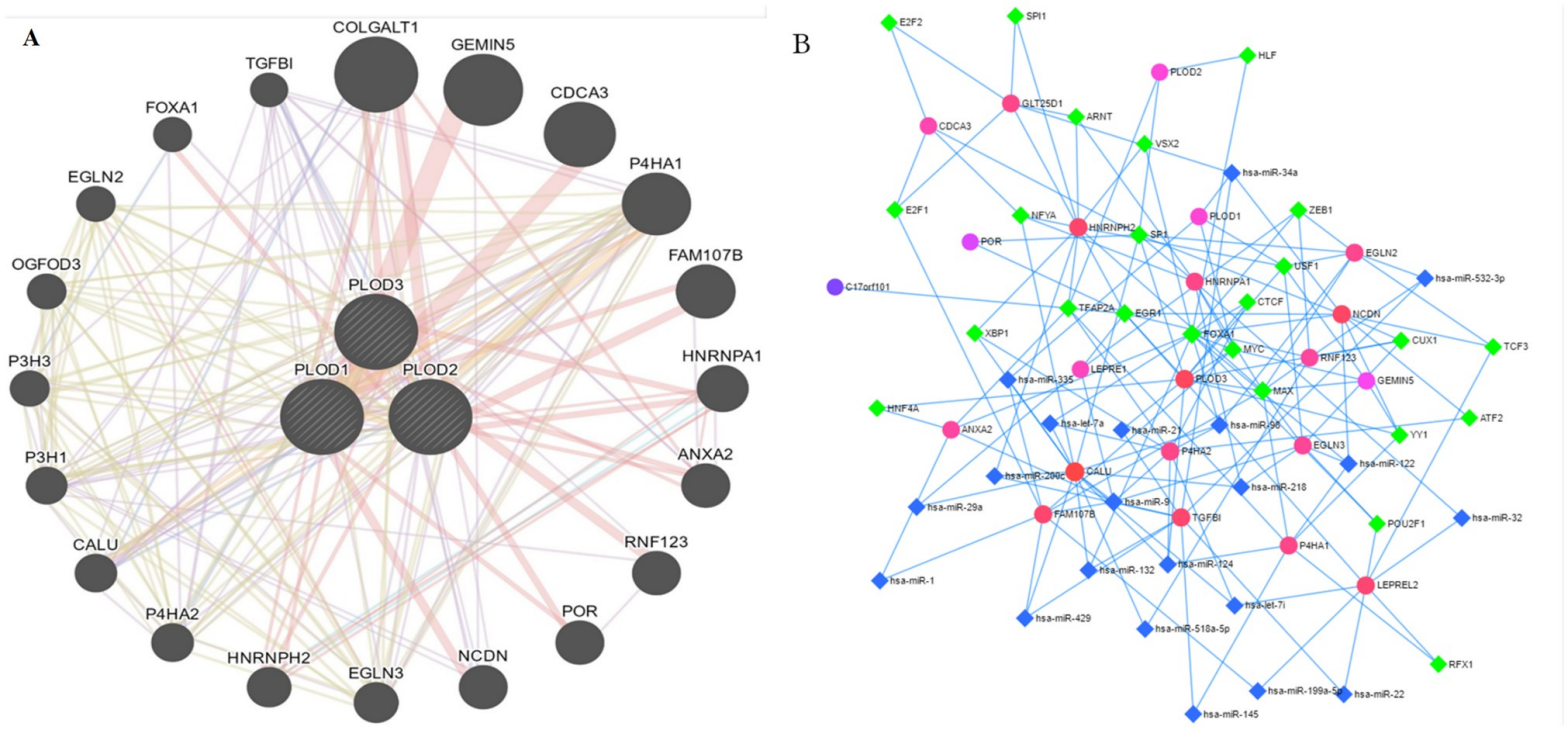
Gene-gene network and miRNA/TF prediction of PLOD family members in LGG. (A) Gene-gene interaction network among PLOD family members. Each node represents a gene. The node size represents the strength of interactions, and the line color represents the types of interactions. (B) Transcription factor-miRNA (TF-miRNA) coregulatory network of significantly PLODs correlated genes.

Moreover, **GeneMANIA** is used to build networks of PLODs family members and their interactive genes. The online tool identified twenty genes closely related to PLODs (Fig 4A). After that, we built up the PLODs family members related TF-miRNA regulatory network, Fig 4B shows the regulation network composed of miRNAs and target genes, including 23 seeds, 155 edges and 67 nodes (S1 File).

In short, these interactive genes and miRNAs may be potential targets for LGG formation and deserve further study.

### GO enrichment and KEGG analysis of PLOD1/2/3

**Fig 5:**
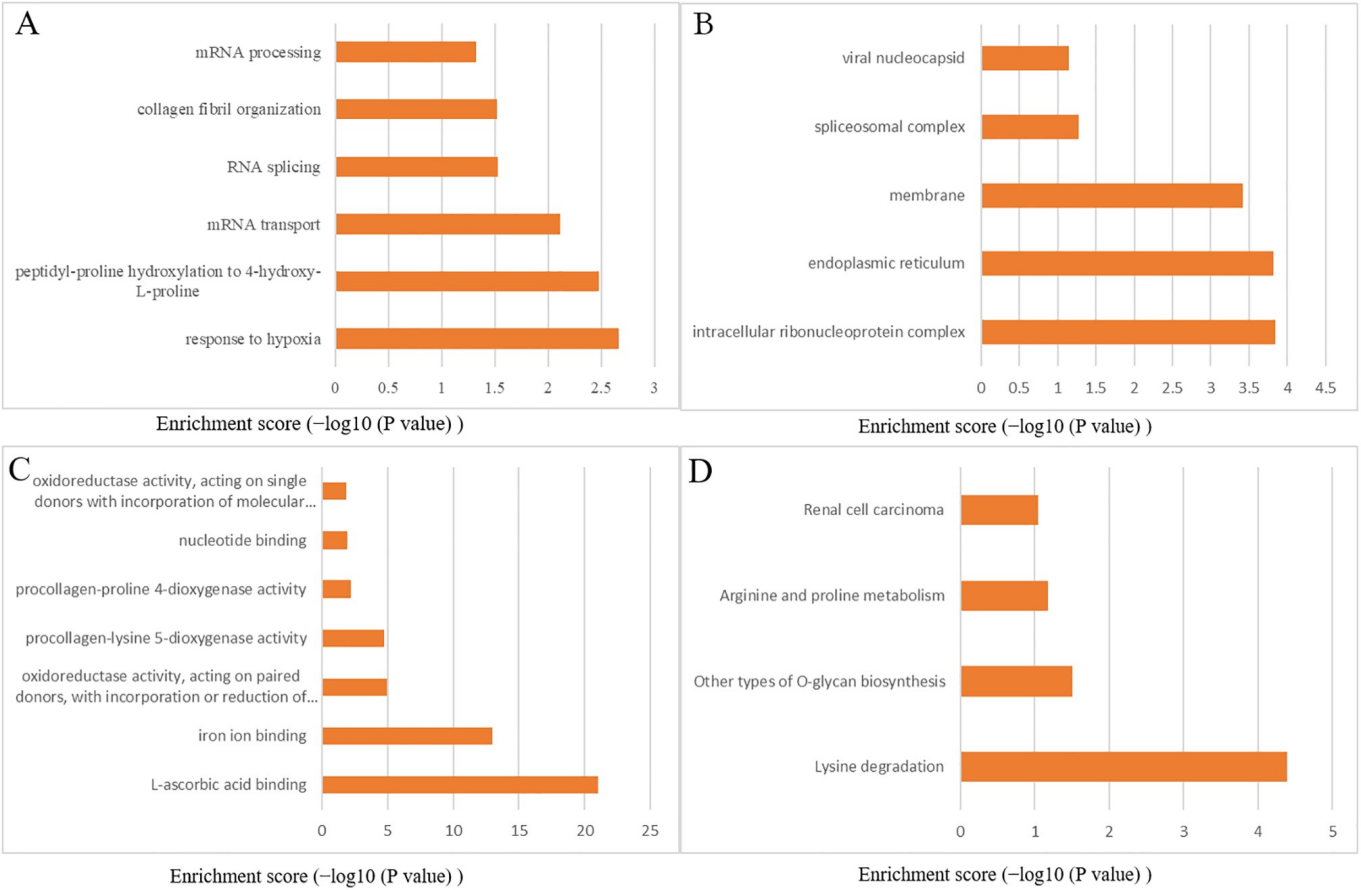
GO annotation and KEGG pathway enrichment analysis of PLOD family members in LGG. The top enriched GO (A) BP, (B) CC and (C) MF terms as well (D) KEGG pathways. GO, gene ontology; KEGG, Kyoto encyclopedia of genes and genomes; BP, biological process; CC, cellular component; MF, molecular function.

In order to further explore the biological functions that these interactive genes of PLODs in LGG, we used DAVID to construct GO and the KEGG pathway. The results of biological processes (BP) showed that these genes are primarily involved in response to hypoxia, proline hydroxylation to 4-hydroxy-L-proline, mRNA transport, RNA splicing, collagen fibril organization and mRNA processing (Fig 5A). For GO cellular component (CC) analysis, the significantly enriched terms were intracellular ribonucleoprotein complex, endoplasmic reticulum, membrane, spliceosomal complex and viral nucleocapsid (Fig 5B). Significantly enriched molecular function (MF) terms included L-ascorbic acid binding, oxidoreductase activity, procollagen-lysine 5-dioxygenase activity, nucleotide binding and procollagen-proline 4-dioxygenase activity (Fig 5C). KEGG pathway analysis showed enrichment in Lysine degradation, other types of O-glycan biosynthesis, Arginine and proline metabolism, renal cell carcinoma (Fig 5D).

To sum up, the results illustrated that PLODs were mainly engaged in tumor-related regulatory mechanisms, such as response to hypoxia, oxidoreductase activity and Lysine degradation.

### The expression of PLOD family members is correlated with immune infiltration levels in LGG

**Fig 6:**
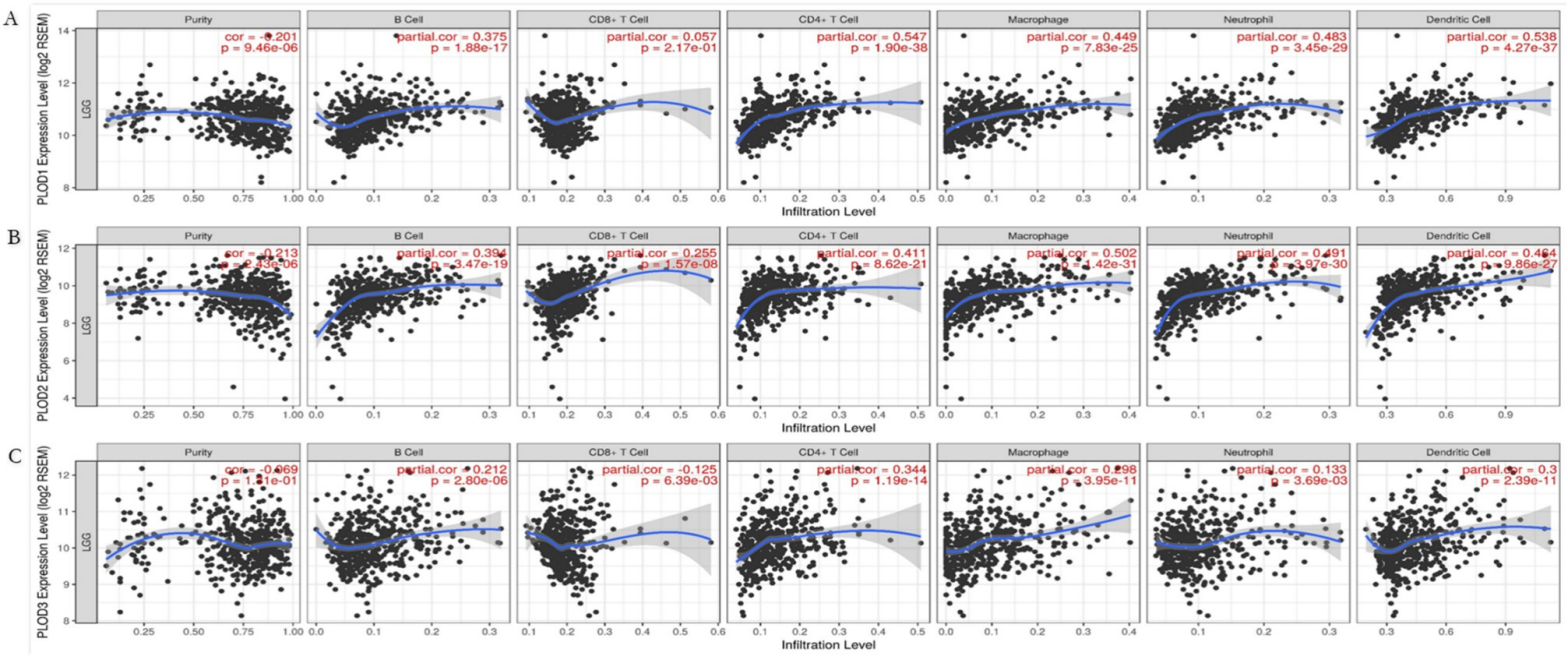
Correlations of PLODs expression with immune infiltration level in LGG. (A) The correlation between each type of TIICs (B-cells, CD4+ T-cells, CD8+ T-cells, neutrophils, macrophages and dendritic cells) and PLOD1. (B) The correlation between each type of TIICs (B-cells, CD4+ T-cells, CD8+ T-cells, neutrophils, macrophages and dendritic cells) and PLOD2. (C) The correlation between each type of TIICs (B-cells, CD4+ T-cells, CD8+ T-cells, neutrophils, macrophages and dendritic cells) and PLOD3.

To explore the immune microenvironment, the relationship of the levels of immune infiltration and the expression of PLODs in LGG was analyzed by TIMER database. The results showed that all the PLODs family members were associated with negative tumor purity. The expression level of PLOD1/2 was significantly positively correlated with the infiltration levels of B cells, CD4 + T cells, CD8 + T cells, neutrophils, macrophages and dendritic cells. Similarly, PLOD3 mRNA expression was positively correlated with infiltrating levels of CD4+ T-cells, neutrophils, macrophages and dendritic cells, except CD8+ T-cells and B-cells (Fig 6).

Taken together, these results further confirm the key role of PLOD family members in regulating immune activity in the LGG microenvironment.

## Discussion

Low-grade gliomas are infiltrating primary central nervous tumor that most commonly occurs in young patients [27] and cannot be cured by the traditional treatment, such as surgery, radiology or a combined approach [28]. Therefore, new biomarkers are urgently to be discovered, which can promote early diagnose and predict the prognosis of LGG patients [29]. Recent research shows that the functions of PLODs have involved in the tumorigenesis and the prognosis of a lot of cancers, yet functions of PLODs in LGGs are still unclear [30–32]. In this manuscript, we analyzed the expression level and prognostic value of PLOD family members in LGGs, with the purpose of proposing new diagnostic and therapeutic strategy for LGG patients.

PLOD1 has been reported that its aberrant expression level was significantly correlated with various human cancers, including prostate cancer, gastric cancer, colorectal cancer and bladder cancer [33–36]. Moreover, previous research has presented that the expression PLOD1 can be predicted prognosis of IDH^mut^ glioma patients, which involved in oxygen metabolism [37]. However, the prognostic significance of PLOD1 in LGG patients has not been reported. In our research, it was also found that the expression level of PLOD1 in LGG tissues was up-regulate than normal brain tissues and was significantly related with poorer prognosis in LGG patients.

PLOD2 has been reported up-regulated in many tumors, which affect collagen remodeling through affecting HIF-1α, TGF-β and microRNA-26a/b [38–40]. In breast cancer tumorigenesis, high expression of PLOD2 was positively associated with poorer prognosis [41]. In the central nervous system, some researchers have reported that hypoxia-induced PLOD2 can promote tumorigenesis via PI3K/Akt signaling in glioma [42]. In addition, Li X et al [43], indicated that knockdown of PLOD2 in glioblastoma (GBM) can play antitumor effect under hypoxia conditions. Up to now, the potential effects of PLOD2 on LGG tumorigenesis remain limited. In this manuscript, PLOD2 was validated a prognostic indicator in patients with LGG, and PLOD2 of significantly overexpressed in LGG tissues.

PLOD3 was also founded in many human solid tumors, containing gastric cancer, lung cancer and hepatocellular cancer [44–46]. In brain tumor, PLOD3 was founded play considerable roles in the proliferation and metastasis of glioblastoma [47].

This research has systematically provided evidence that PLODs may be prognostic factors in the outcome of LGG patients. The results also used multiple bioinformatic platform to validate the over expression levels of PLODs in LGG. The limitation of this research was lack of laboratory experiment procedures and the exact mechanism should be further investigated in further studies. The research showed that PLODs were up-regulated in LGG and can be set as survival risks for this cancer type. To further investigate these genes relationship with immune cells, we predicted gene-immune cell interactions. It is reasonable that higher expression of PLODs is correlated with lower tumor purity and higher immune cells infiltration. In our research, PLOD family genes had diverse correlation coefficients with different immune cells, presenting the diverse function of genes in immune infiltration and tumor-immune interplay. This may suggest the immune infiltration possibly slows down tumor growth and metastases through its specific ways. But future experimental researches are needed to confirm their functions and interplays.

Previous studies revealed that the mechanisms of PLODs in cancer development mainly involved in regulating the collagen metabolism, hypoxia, extracellular matrix construction, and immune microenviroment [10, 34]. In our research, the mutation of PLODs in LGG was founded and closely related to the prognosis of LGG patients. But we have not studied the relationship between special types of mutations and the prognosis of gliomas, such as functional mutations, hot regions and mutational pattern. With the gradual improvement of genomics information, we will continue to study this part of the content in future research. And we also found 20 genes related to the plod family and constructed the gene network, among which COLGALT1(collagen beta(1-O) galactosyltransferase type 1) is the most closely related gene. In the previous research, it was found that COLGALT1 a variety of biological processes such as cell attachment, migration, proliferation, and differentiation [48]. However, its relationship with the occurrence and development of tumors is still unclear, and needs to be further studied, especially LGG. In this study, GO and KEGG pathway enrichment analysis was performed to understand the biological functions of PLODs in LGG, mainly involved in hypoxia, oxidoreductase activity and Lysine degradation. The results illustrated the mechanisms by which PLODs are regulating tumorigenesis. In the recent clinical studies, some immune therapy for LGG has broad prospects, such as CAR-T cell therapy [49]. We interestingly founded that the expression of PLODs was closely related with tumor purity and immune cell infiltration, which may be insight biomarkers for immune therapy for LGG.

## Conclusions

This is the first study to our knowledge to investigate the relationship between the expression of all PLOD family genes in LGG and patient outcomes. Taken together, this study strongly suggests that PLOD family members may be potential therapeutic targets and serve as prognostic markers for LGG patients’ survival.

## Supporting information

S1 File (ZIP)

## References

[1] Morshed RA, Young JS, Hervey-Jumper SL, Berger MS. The management of low-grade gliomas in adults. J Neurosurg Sci. 2019;63(4):450–7. 10.23736/S0390-5616.19.04701-5 30916536

[2] Masui K, Cavenee WK, Mischel PS. Cancer metabolism as a central driving force of glioma pathogenesis. Brain Tumor Pathol. 2016;33(3):161–8. 10.1007/s10014-016-0265-5 27295313 PMC5488809

[3] Bernstock JD, Mooney JH, Ilyas A, Chagoya G, Estevez-Ordonez D, Ibrahim A, et al Molecular and cellular intratumoral heterogeneity in primary glioblastoma: clinical and translational implications. J Neurosurg. 2019:1–9. 10.3171/2019.5.JNS19364 31443071

[4] Zhou Y, Wu W, Bi H, Yang D, Zhang C. Glioblastoma precision therapy: From the bench to the clinic. Cancer Lett. 2020;475:79–91. 10.1016/j.canlet.2020.01.027 32004571

[5] Heikkinen J, Risteli M, Wang C, Latvala J, Rossi M, Valtavaara M, et al Lysyl hydroxylase 3 is a multifunctional protein possessing collagen glucosyltransferase activity. The Journal of biological chemistry. 2000;275(46):36158–63. 10.1074/jbc.M006203200 10934207

[6] Salo AM, Cox H, Farndon P, Moss C, Grindulis H, Risteli M, et al A connective tissue disorder caused by mutations of the lysyl hydroxylase 3 gene. Am J Hum Genet. 2008;83(4):495–503. 10.1016/j.ajhg.2008.09.004 18834968 PMC2561927

[7] Scietti L, Campioni M, Forneris F. SiMPLOD, a Structure-Integrated Database of Collagen Lysyl Hydroxylase (LH/PLOD) Enzyme Variants. J Bone Miner Res. 2019;34(7):1376–82. 10.1002/jbmr.3692 30721533

[8] Rautavuoma K, Takaluoma K, Sormunen R, Myllyharju J, Kivirikko KI, Soininen R. Premature aggregation of type IV collagen and early lethality in lysyl hydroxylase 3 null mice. Proc Natl Acad Sci U S A. 2004;101(39):14120–5. 10.1073/pnas.0404966101 15377789 PMC521128

[9] Ah-Kim H, Zhang X, Islam S, Sofi JI, Glickberg Y, Malemud CJ, et al Tumour necrosis factor alpha enhances the expression of hydroxyl lyase, cytoplasmic antiproteinase-2 and a dual specificity kinase TTK in human chondrocyte-like cells. Cytokine. 2000;12(2):142–50. 10.1006/cyto.1999.0539 10671299

[10] Qi Y, Xu R. Roles of PLODs in Collagen Synthesis and Cancer Progression. Front Cell Dev Biol. 2018;6:66 10.3389/fcell.2018.00066 30003082 PMC6031748

[11] Rhodes DR, Kalyana-Sundaram S, Mahavisno V, Varambally R, Yu J, Briggs BB, et al Oncomine 3.0: genes, pathways, and networks in a collection of 18,000 cancer gene expression profiles. Neoplasia. 2007;9(2):166–80. 10.1593/neo.07112 17356713 PMC1813932

[12] Uhlen M, Fagerberg L, Hallstrom BM, Lindskog C, Oksvold P, Mardinoglu A, et al Proteomics. Tissue-based map of the human proteome. Science (New York, NY). 2015;347(6220):1260419 10.1126/science.1260419 25613900

[13] Uhlén M, Fagerberg L, Hallström BM, Lindskog C, Oksvold P, Mardinoglu A, et al Proteomics. Tissue-based map of the human proteome. Science (New York, NY). 2015;347(6220):1260419 10.1126/science.1260419 25613900

[14] Tang Z, Li C, Kang B, Gao G, Li C, Zhang Z. GEPIA: a web server for cancer and normal gene expression profiling and interactive analyses. Nucleic Acids Res. 2017;45(W1):W98–w102. 10.1093/nar/gkx247 28407145 PMC5570223

[15] Gao J, Aksoy BA, Dogrusoz U, Dresdner G, Gross B, Sumer SO, et al Integrative analysis of complex cancer genomics and clinical profiles using the cBioPortal. Sci Signal. 2013;6(269):pl1 10.1126/scisignal.2004088 23550210 PMC4160307

[16] Warde-Farley D, Donaldson SL, Comes O, Zuberi K, Badrawi R, Chao P, et al The GeneMANIA prediction server: biological network integration for gene prioritization and predicting gene function. Nucleic Acids Res. 2010;38(Web Server issue):W214–20. 10.1093/nar/gkq537 20576703 PMC2896186

[17] Zhou G, Soufan O, Ewald J, Hancock REW, Basu N, Xia J. NetworkAnalyst 3.0: a visual analytics platform for comprehensive gene expression profiling and meta-analysis. Nucleic Acids Res. 2019;47(W1):W234–w41. 10.1093/nar/gkz240 30931480 PMC6602507

[18] Huang da W, Sherman BT, Lempicki RA. Systematic and integrative analysis of large gene lists using DAVID bioinformatics resources. Nat Protoc. 2009;4(1):44–57. 10.1038/nprot.2008.211 19131956

[19] Li T, Fan J, Wang B, Traugh N, Chen Q, Liu JS, et al TIMER: A Web Server for Comprehensive Analysis of Tumor-Infiltrating Immune Cells. Cancer Res. 2017;77(21):e108–e10. 10.1158/0008-5472.CAN-17-0307 29092952 PMC6042652

[20] Bredel M, Bredel C, Juric D, Harsh GR, Vogel H, Recht LD, et al Functional network analysis reveals extended gliomagenesis pathway maps and three novel MYC-interacting genes in human gliomas. Cancer Res. 2005;65(19):8679–89. 10.1158/0008-5472.CAN-05-1204 16204036

[21] Sun L, Hui AM, Su Q, Vortmeyer A, Kotliarov Y, Pastorino S, et al Neuronal and glioma-derived stem cell factor induces angiogenesis within the brain. Cancer Cell. 2006;9(4):287–300. 10.1016/j.ccr.2006.03.003 16616334

[22] Rickman DS, Bobek MP, Misek DE, Kuick R, Blaivas M, Kurnit DM, et al Distinctive molecular profiles of high-grade and low-grade gliomas based on oligonucleotide microarray analysis. Cancer Res. 2001;61(18):6885–91. 11559565

[23] Shai R, Shi T, Kremen TJ, Horvath S, Liau LM, Cloughesy TF, et al Gene expression profiling identifies molecular subtypes of gliomas. Oncogene. 2003;22(31):4918–23. 10.1038/sj.onc.1206753 12894235

[24] Liang Y, Diehn M, Watson N, Bollen AW, Aldape KD, Nicholas MK, et al Gene expression profiling reveals molecularly and clinically distinct subtypes of glioblastoma multiforme. Proc Natl Acad Sci U S A. 2005;102(16):5814–9. 10.1073/pnas.0402870102 15827123 PMC556127

[25] French PJ, Swagemakers SM, Nagel JH, Kouwenhoven MC, Brouwer E, van der Spek P, et al Gene expression profiles associated with treatment response in oligodendrogliomas. Cancer Res. 2005;65(24):11335–44. 10.1158/0008-5472.CAN-05-1886 16357140

[26] Lee J, Kotliarova S, Kotliarov Y, Li A, Su Q, Donin NM, et al Tumor stem cells derived from glioblastomas cultured in bFGF and EGF more closely mirror the phenotype and genotype of primary tumors than do serum-cultured cell lines. Cancer Cell. 2006;9(5):391–403. 10.1016/j.ccr.2006.03.030 16697959

[27] Kumthekar P, Raizer J, Singh S. Low-grade glioma. Cancer Treat Res. 2015;163:75–87. 10.1007/978-3-319-12048-5_5 25468226

[28] Laviv Y. [DIFFUSE LOW GRADE GLIOMA: PERSONALIZED ADAPTATION OF SURGICAL RESECTION IN AN ERA OF TARGETED ONCOLOGICAL THERAPY]. Harefuah. 2019;158(9):601–6. 31507113

[29] Kim B, Tabori U, Hawkins C. An update on the CNS manifestations of brain tumor polyposis syndromes. Acta Neuropathol. 2020 10.1007/s00401-020-02124-y 31970492

[30] Gjaltema RA, de Rond S, Rots MG, Bank RA. Procollagen Lysyl Hydroxylase 2 Expression Is Regulated by an Alternative Downstream Transforming Growth Factor beta-1 Activation Mechanism. The Journal of biological chemistry. 2015;290(47):28465–76. 10.1074/jbc.M114.634311 26432637 PMC4653703

[31] Shen Q, Eun JW, Lee K, Kim HS, Yang HD, Kim SY, et al Barrier to autointegration factor 1, procollagen-lysine, 2-oxoglutarate 5-dioxygenase 3, and splicing factor 3b subunit 4 as early-stage cancer decision markers and drivers of hepatocellular carcinoma. Hepatology. 2018;67(4):1360–77. 10.1002/hep.29606 29059470

[32] Li L, Wang W, Li X, Gao T. Association of ECRG4 with PLK1, CDK4, PLOD1 and PLOD2 in esophageal squamous cell carcinoma. Am J Transl Res. 2017;9(8):3741–8. 28861165 PMC5575188

[33] Ross JA, Vissers JPC, Nanda J, Stewart GD, Husi H, Habib FK, et al The influence of hypoxia on the prostate cancer proteome. Clin Chem Lab Med. 2020 10.1515/cclm-2019-0626 31940282

[34] Li SS, Lian YF, Huang YL, Huang YH, Xiao J. Overexpressing PLOD family genes predict poor prognosis in gastric cancer. J Cancer. 2020;11(1):121–31. 10.7150/jca.35763 31892979 PMC6930397

[35] Boonsongserm P, Angsuwatcharakon P, Puttipanyalears C, Aporntewan C, Kongruttanachok N, Aksornkitti V, et al Tumor-induced DNA methylation in the white blood cells of patients with colorectal cancer. Oncol Lett. 2019;18(3):3039–48. 10.3892/ol.2019.10638 31452782 PMC6676401

[36] Yamada Y, Kato M, Arai T, Sanada H, Uchida A, Misono S, et al Aberrantly expressed PLOD1 promotes cancer aggressiveness in bladder cancer: a potential prognostic marker and therapeutic target. Mol Oncol. 2019;13(9):1898–912. 10.1002/1878-0261.12532 31199049 PMC6717764

[37] Dao Trong P, Rosch S, Mairbaurl H, Pusch S, Unterberg A, Herold-Mende C, et al Identification of a Prognostic Hypoxia-Associated Gene Set in IDH-Mutant Glioma. Int J Mol Sci. 2018;19(10). 10.3390/ijms19102903 30257451 PMC6212863

[38] Du H, Pang M, Hou X, Yuan S, Sun L. PLOD2 in cancer research. Biomed Pharmacother. 2017;90:670–6. 10.1016/j.biopha.2017.04.023 28415047

[39] Cao F, Kang XH, Cui YH, Wang Y, Zhao KL, Wang YN, et al [Upregulation of PLOD2 promotes invasion and metastasis of osteosarcoma cells]. Zhonghua Zhong Liu Za Zhi. 2019;41(6):435–40. 10.3760/cma.j.issn.0253-3766.2019.06.007 31216829

[40] Chen P, Gu YY, Ma FC, He RQ, Li ZY, Zhai GQ, et al Expression levels and cotargets of miRNA1263p and miRNA1265p in lung adenocarcinoma tissues: Alphan exploration with RTqPCR, microarray and bioinformatic analyses. Oncol Rep. 2019;41(2):939–53. 10.3892/or.2018.6901 30535503 PMC6313014

[41] Hu HL, Wang CF, Wei XH, Lv JX, Cao XH, Shi YY, et al Correlation between procollagen-lysine, 2-oxoglutarate 5-dioxygenase 2 and breast cancer. Int J Clin Exp Pathol. 2019;12(3):1015–21. 31933913 PMC6945164

[42] Song Y, Zheng S, Wang J, Long H, Fang L, Wang G, et al Hypoxia-induced PLOD2 promotes proliferation, migration and invasion via PI3K/Akt signaling in glioma. Oncotarget. 2017;8(26):41947–62. 10.18632/oncotarget.16710 28410212 PMC5522040

[43] Xu Y, Zhang L, Wei Y, Zhang X, Xu R, Han M, et al Procollagen-lysine 2-oxoglutarate 5-dioxygenase 2 promotes hypoxia-induced glioma migration and invasion. Oncotarget. 2017;8(14):23401–13. 10.18632/oncotarget.15581 28423580 PMC5410313

[44] Wang B, Xu L, Ge Y, Cai X, Li Q, Yu Z, et al PLOD3 is Upregulated in Gastric Cancer and Correlated with Clinicopathologic Characteristics. Clin Lab. 2019;65(1). 10.7754/Clin.Lab.2018.180541 30775879

[45] Baek JH, Yun HS, Kwon GT, Kim JY, Lee CW, Song JY, et al PLOD3 promotes lung metastasis via regulation of STAT3. Cell Death Dis. 2018;9(12):1138 10.1038/s41419-018-1186-5 30442941 PMC6237925

[46] Lin Y, Liang R, Ye J, Li Q, Liu Z, Gao X, et al A twenty gene-based gene set variation score reflects the pathological progression from cirrhosis to hepatocellular carcinoma. Aging (Albany NY). 2019;11(23):11157–69. 10.18632/aging.102518 31811111 PMC6932912

[47] Tsai CK, Huang LC, Tsai WC, Huang SM, Lee JT, Hueng DY. Overexpression of PLOD3 promotes tumor progression and poor prognosis in gliomas. Oncotarget. 2018;9(21):15705–20. 10.18632/oncotarget.24594 29644003 PMC5884658

[48] Baumann S, Hennet T. Collagen Accumulation in Osteosarcoma Cells lacking GLT25D1 Collagen Galactosyltransferase. The Journal of biological chemistry. 2016;291(35):18514–24. 10.1074/jbc.M116.723379 27402836 PMC5000096

[49] Zhu H, You Y, Shen Z, Shi L. EGFRvIII-CAR-T Cells with PD-1 Knockout Have Improved Anti-Glioma Activity. Pathol Oncol Res. 2020 10.1007/s12253-019-00759-1 31989402

