# Supplementary material for: Comprehensive analysis of PLOD family members in low-grade gliomas using bioinformatics methods
Source: PLoS One. 2021 Jan 27;16(1):e0246097. doi: 10.1371/journal.pone.0246097 (PMC7840023; doi:10.1371/journal.pone.0246097)
Supplement: S1 File — (ZIP) [file pone.0246097.s001.zip › Supplement 1/Supplement for GeneMINIA network/genemania-report.pdf]

GeneMANIA report

Created on : 17 October 2020 11:57:08
Last database update : 13 March 2017 00:00:00
Application version : 3.6.0

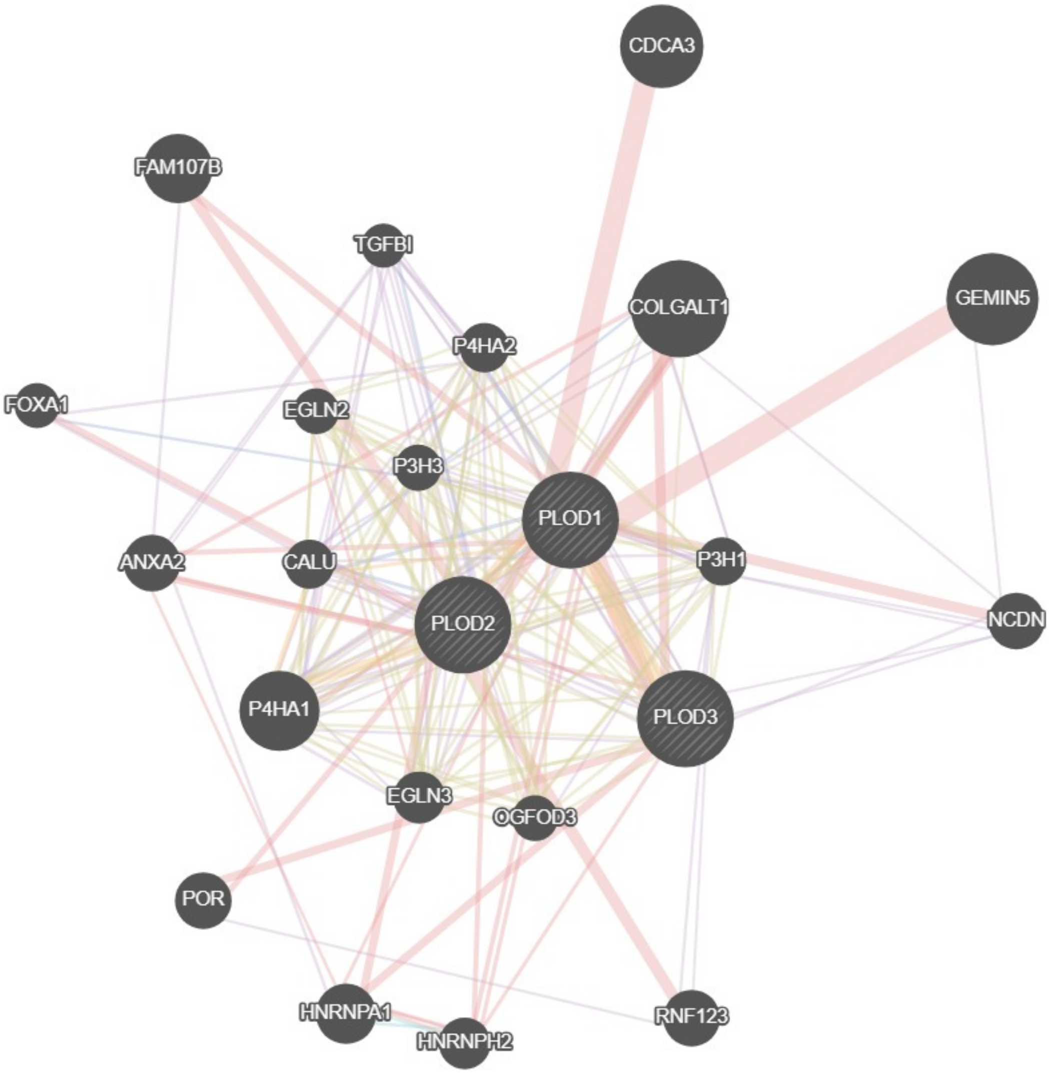

Networks

- Physical Interactions
- Co-expression
- Predicted
- Co-localization
- Pathway
- Genetic Interactions
- Shared protein domains

Functions

N/A

Search parameters

Organism Homo sapiens (human)

Genes PLOD1 , PLOD3 , PLOD2

Network weighting Automatically selected weighting method

Networks

A

Abu-Odeh-Aqeilan-2014 , Agrawal-Sedivy-2010 , Aichem-Groettrup-2012 , Albers-Koegl-2005 , Alexandru-Deshaies-2008 , Alizadeh-Staudt-2000 , Andresen-Flores-Morales-2014 , Arbuckle-Grant-2010 , Arroyo-Aloy-2014 , Arroyo-Aloy-2015

B

Bahr-Bowler-2013 , Bailey-Hieter-2015 , Bandyopadhyay-Ideker-2010 , Bantscheff-Drewes-2011 , Barr-Knapp-2009 , Barrios-Rodiles-Wrana-2005 , Behrends-Harper-2010 , Behzadnia-Lührmann-2007 , Bennett-Harper-2010 , Benzinger-Hermeking-2005 , Berggård-James-2006 , Bett-Hay-2013 , Bhatnagar-Attie-2014 , Bild-Nevins-2006 B , BIOGRID-SMALL-SCALE-STUDIES , BIOGRID-SMALL-SCALE-STUDIES , Blandin-Richard-2013 , Blomen-Brummelkamp-2015 , Blomen-Brummelkamp-2015 , Bogachek-Weigel-2014 , Boldrick-Relman-2002 , Bonacci-Soubeyran-2014 , Bouwmeester-Superti-Furga-2004 , Brajenovic-Drewes-2004 , Brehme-Superti-Furga-2009 , Bruderer-Hay-2011 , Burington-Shaughnessy-2008 , Butland-Hayden-2014 , Byron-Humphries-2012

C

Cai-Conaway-2007 , Camargo-Brandon-2007 , Campos-Reinberg-2015 , Cao-Chinnaiyan-2014 , Carmon-Liu-2014 , CELL_MAP , Chen-Brown-2002 , Chen-Ge-2013 , Chen-Huang-2014 , Chen-Zhang-2013 , Christianson-Kopito-2011 , Cloutier-Coulombe-2013 , Colland-Gauthier-2004 , Corominas-Iakoucheva-2014 , Couzens-Gingras-2013 , Cox-Rizzino-2013 , Coyaud-Raught-2015

D

Danielsen-Nielsen-2011 , Dart-Wells-2015 , de Hoog-Mann-2004 , Diner-Cristea-2015 , Dobbin-Giordano-2005 , Drissi-Boisvert-2015 , Dyer-Sobral-2010

E

Emanuele-Elledge-2011 , Emdal-Olsen-2015 , Ewing-Figeys-2007

F

Fenner-Prehn-2010 , Floyd-Pagliarini-2016 , Foerster-Ritter-2013 , Fogeron-Lange-2013 , Foster-Marshall-2013 , Freibaum-Taylor-2010

G

Gabriel-Baumgrass-2016 , Galligan-Howley-2015 , Gao-Reinberg-2012 , Gautier-Hall-2009 , Giannone-Liu-2010 , Glatter-Gstaiger-2009 , Gloeckner-Ueffing-2007 ,

G

Goehler-Wanker-2004 , Golebiowski-Hay-2009 , Goudreault-Gingras-2009 , Grant-2010 , Greco-Cristea-2011 , Grossmann-Stelzl-2015 , Guarani-Harper-2014 , Gupta-Pelletier-2015

H

Hanson-Clayton-2014 , Hauri-Gstaiger-2013 , Havrylov-Redowicz-2009 , Havugimana-Emili-2012 , Hayes-Urbé-2012 , Hegele-Stelzl-2012 A , Hegele-Stelzl-2012 B , Hein-Mann-2015 , Hill-Livingston-2014 , HUMANCYC , Humphries-Humphries-2009 , Hutchins-Peters-2010 , Huttlin-Gygi-2015

I

I2D-BIND-Fly2Human , I2D-BIND-Mouse2Human , I2D-BIND-Rat2Human , I2D-BIND-Worm2Human , I2D-BIND-Yeast2Human , I2D-BioGRID-Fly2Human , I2D-BioGRID-Mouse2Human , I2D-BioGRID-Rat2Human , I2D-BioGRID-Worm2Human , I2D-BioGRID-Yeast2Human , I2D-Chen-Pawson-2009-PiwiScreen-Mouse2Human , I2D-Formstecher-Daviet-2005-Embryo-Fly2Human , I2D-Giot-Rothbert-2003-Low-Fly2Human , I2D-INNATEDB-Mouse2Human , I2D-IntAct-Fly2Human , I2D-IntAct-Mouse2Human , I2D-IntAct-Rat2Human , I2D-IntAct-Worm2Human , I2D-IntAct-Yeast2Human , I2D-Krogan-Greenblatt-2006-Core-Yeast2Human , I2D-Krogan-Greenblatt-2006-NonCore-Yeast2Human , I2D-Li-Vidal-2004-CORE-1-Worm2Human , I2D-Li-Vidal-2004-non-core-Worm2Human , I2D-Manual-Mouse2Human , I2D-Manual-Rat2Human , I2D-MGI-Mouse2Human , I2D-MINT-Fly2Human , I2D-MINT-Mouse2Human , I2D-MINT-Rat2Human , I2D-MINT-Worm2Human , I2D-MINT-Yeast2Human , I2D-Ptacek-Snyder-2005-Yeast2Human , I2D-Tarassov-PCA-Yeast2Human , I2D-Tewari-Vidal-2004-TGFb-Worm2Human , I2D-vonMering-Bork-2002-High-Yeast2Human , I2D-vonMering-Bork-2002-Low-Yeast2Human , I2D-vonMering-Bork-2002-Medium-Yeast2Human , I2D-Wang-Orkin-2006-EScmplx-Mouse2Human , I2D-Wang-Orkin-2006-EScmplxlow-Mouse2Human , I2D-Yu-Vidal-2008-GoldStd-Yeast2Human , IMID , Ingham-Pawson-2005 , Innocenti-Brown-2011 , INTERPRO , IREF-BIND , IREF-BIOGRID , IREF-DIP , IREF-HPRD , IREF-INTACT , IREF-MATRIXDB , IREF-MPPI , IREF-PUBMED , IREF-SMALL-SCALE-STUDIES , IREF-SMALL-SCALE-STUDIES

J

Jeronimo-Coulombe-2007 , Jin-Pawson-2004 , Johnson-Kerner-Wichterle-2015 , Johnson-Shoemaker-2003 , Jones-MacBeath-2006 , Joshi-Cristea-2013 , Jäger-Krogan-2011

K

Kahle-Zoghbi-2011 , Kaltenbach-Hughes-2007 , Katsogiannou-Rocchi-2014 , Kim-Gygi-2011 , Kim-Major-2015 , Kneissl-Grummt-2003 , Koch-Hermeking-2007 , Kotlyar-Jurisica-2015 , Kristensen-Foster-2012 , Kärblane-Sarmiento-2015 , Kırlı-Görlich-2015

L

Lambert-Gingras-2015 , Lamoliatte-Thibault-2014 , Lau-Ronai-2012 , Lee-Songyang-2011 , Lehner-Sanderson-2004 A , Lehner-Sanderson-2004 B , Leng-Wang-2014 , Leung-Jones-2014 , Li-Chen-2015 , Li-Dorf-2011 A , Li-Dorf-2011 B , Li-Dorf-2014 , Li-Haura-2013 , Lim-Zoghbi-2006 , Lin-Smith-2010 , Lipp-Guthrie-2015 , Liu-Wang-2012 , Llères-Lamond-2010 , Loch-Strickler-2012 , Low-Heck-2014 , Lu-Zhang-2013 , Luo-Elledge-2009

M

Mak-Moffat-2010 , Mallon-McKay-2013 , Malovannaya-Qin-2010 , Markson-Sanderson-2009 , Maréchal-Zou-2014 , Matsumoto-Nakayama-2005 , McCracken-Blencowe-2005 , McFarland-Nussbaum-2008 , Meek-Piwnica-Worms-2004 , Milev-Mouland-2012 , Miyamoto-Sato-Yanagawa-2010 , Murakawa-Landthaler-2015

N

Nakayama-Ohara-2002 , Nakayasu-Adkins-2013 , Napolitano-Meroni-2011 , Narayan-Bennett-2012 , Nathan-Goldberg-2013 , NCI_NATURE , Neganova-Lako-2011 , Newman-Keating-2003 , Nicholson-Hupp-2014 , Noble-Diehl-2008

O

Oliviero-Cagney-2015 , Olma-Pintard-2009 , Oláh-Ovádi-2011 , Oshikawa-Nakayama-2012 , Ouyang-Gill-2009

P

Panigrahi-Pati-2012 , Papp-Lamia-2015 , Perez-Hernandez-Yáñez-Mó-2013 , Perou-Botstein-1999 , Perou-Botstein-2000 , Persaud-Rotin-2009 , Petschnigg-Stagljar-2014 , PFAM , Phillips-Corn-2013 , Pichlmair-Superti-Furga-2011 , Pichlmair-Superti-Furga-2012 , Pilot-Storck-Goillot-2010 , Povlsen-Choudhary-2012

R

Ramachandran-LaBaer-2004 , Raman-Harper-2015 , Ramaswamy-Golub-2001 , Ravasi-Hayashizaki-2010 , REACTOME , Reinke-Keating-2013 , Reyniers-Taymans-2014 , Richter-Chrzanowska-Lightowlers-2010 , Rieger-Chu-2004 , Rolland-Vidal-2014 , Rosenwald-Staudt-2001 , Roth-Zlotnik-2006 , Roux-Burke-2012 , Rowbotham-Mermoud-2011 , Roy-Pardo-2014 , Roy-Parent-2013 , Rual-Vidal-2005 A , Rual-Vidal-2005 B

S

Sang-Jackson-2011 , Sato-Conaway-2004 , Schadt-Shoemaker-2004 , Scholz-Taylor-2016 , Singh-Moore-2012 , Smirnov-Cheung-2009 , So-Colwill-2015 , Soler-López-Aloy-2011 , Sowa-Harper-2009 , Stehling-Lill-2012 , Stehling-Lill-2013 , Stelzl-Wanker-2005 , Stes-Gevaert-2014 , Stuart-Kim-2003 , Suter-Wanker-2013

T

Taipale-Lindquist-2012 , Taipale-Lindquist-2014 , Takahashi-Conaway-2011 , Tarallo-Weisz-2011 , Tatham-Hay-2011 , Teixeira-Gomes-2010 , Thalappilly-

T

Dusetti-2008 , Thompson-Luchansky-2014 , Tong-Moran-2014 , Toyoshima-Grandori-2012 , Tsai-Cristea-2012

U

Udeshi-Carr-2012

V

van Wijk-Timmers-2009 , Vandamme-Angrand-2011 , Varjosalo-Gstaiger-2013 , Varjosalo-Superti-Furga-2013 , Venkatesan-Vidal-2009 , Vermeulen-Mann-2010 , Vinayagam-Wanker-2011 , Virok-Fülöp-2011 , Vizeacoumar-Moffat-2013

W

Wagner-Choudhary-2011 , Wallach-Kramer-2013 , Wan-Emili-2015 , Wang-Balch-2006 , Wang-Cheung-2015 , Wang-He-2008 , Wang-Maris-2006 , Wang-Xu-2015 , Wang-Yang-2011 , Weimann-Stelzl-2013 A , Weimann-Stelzl-2013 B , Weinmann-Meister-2009 , Wen-Wu-2014 , Whisenant-Salomon-2015 , Wilker-Yaffe-2007 , Willingham-Muchowski-2003 , Witt-Labeit-2008 , Wong-O'Bryan-2012 , Woods-Monteiro-2012 , Woodsmith-Sanderson-2012 , Wu-Garvey-2007 , Wu-Li-2007 , Wu-Ma-2012 , Wu-Stein-2010 , Wu-Stein-2010

X

Xiao-Lefkowitz-2007 , Xie-Cong-2013 , Xie-Green-2012 , Xu-Ye-2012

Y

Yang-Chen-2010 , Yatim-Benkirane-2012 , Yu-Chow-2013 , Yu-Vidal-2011

Z

Zanon-Pichler-2013 , Zhang-Shang-2006 , Zhang-Zou-2011 , Zhao-Krug-2005 , Zhao-Yang-2011 , Zhou-Conrads-2004 , Zhou-Hanemann-2016

Genes

Gene	Description	Rank
PLOD3	procollagen-lysine,2-oxoglutarate 5-dioxygenase 3 [Source:HGNC Symbol;Acc:HGNC:9083]	N/A
PLOD2	procollagen-lysine,2-oxoglutarate 5-dioxygenase 2 [Source:HGNC Symbol;Acc:HGNC:9082]	N/A
PLOD1	procollagen-lysine,2-oxoglutarate 5-dioxygenase 1 [Source:HGNC Symbol;Acc:HGNC:9081]	N/A
COLGALT1	collagen beta(1-O)galactosyltransferase 1 [Source:HGNC Symbol;Acc: HGNC:26182]	1
GEMIN5	gem nuclear organelle associated protein 5 [Source:HGNC Symbol;Acc: HGNC:20043]	2
CDCA3	cell division cycle associated 3 [Source:HGNC Symbol;Acc:HGNC:14624]	3
P4HA1	prolyl 4-hydroxylase subunit alpha 1 [Source:HGNC Symbol;Acc:HGNC: 8546]	4
FAM107B	family with sequence similarity 107 member B [Source:HGNC Symbol; Acc:HGNC:23726]	5
HNRNPA1	heterogeneous nuclear ribonucleoprotein A1 [Source:HGNC Symbol;Acc: HGNC:5031]	6
ANXA2	annexin A2 [Source:HGNC Symbol;Acc:HGNC:537]	7
RNF123	ring finger protein 123 [Source:HGNC Symbol;Acc:HGNC:21148]	8
POR	cytochrome p450 oxidoreductase [Source:HGNC Symbol;Acc:HGNC: 9208]	9
NCDN	neurochondrin [Source:HGNC Symbol;Acc:HGNC:17597]	10
EGLN3	egl-9 family hypoxia inducible factor 3 [Source:HGNC Symbol;Acc: HGNC:14661]	11
HNRNPH2	heterogeneous nuclear ribonucleoprotein H2 (H') [Source:HGNC Symbol; Acc:HGNC:5042]	12
P4HA2	prolyl 4-hydroxylase subunit alpha 2 [Source:HGNC Symbol;Acc:HGNC: 8547]	13
CALU	calumenin [Source:HGNC Symbol;Acc:HGNC:1458]	14
P3H1	prolyl 3-hydroxylase 1 [Source:HGNC Symbol;Acc:HGNC:19316]	15
P3H3	prolyl 3-hydroxylase 3 [Source:HGNC Symbol;Acc:HGNC:19318]	16
OGFOD3	2-oxoglutarate and iron dependent oxygenase domain containing 3 [Source:HGNC Symbol;Acc:HGNC:26174]	17
EGLN2	egl-9 family hypoxia inducible factor 2 [Source:HGNC Symbol;Acc:	18

Gene	Description	Rank
	HGNC:14660]	
FOXA1	forkhead box A1 [Source:HGNC Symbol;Acc:HGNC:5021]	19
TGFBI	transforming growth factor beta induced [Source:HGNC Symbol;Acc: HGNC:11771]	20

Networks

Physical Interactions 67.64%

Matsumoto-Nakayama-2005 5.54%

Large-scale analysis of the human ubiquitin-related proteome. Matsumoto et al (2005). *Proteomics*

Physical Interactions with 311 interactions from BioGRID

Singh-Moore-2012 4.12%

The cellular EJC interactome reveals higher-order mRNP structure and an EJC-SR protein nexus. Singh et al (2012). *Cell*

Physical Interactions with 301 interactions from iRefIndex

Petschnigg-Stagljar-2014 3.90%

The mammalian-membrane two-hybrid assay (MaMTH) for probing membrane-protein interactions in human cells. Petschnigg et al (2014). *Nat Methods*

Physical Interactions with 122 interactions from BioGRID

Whisenant-Salomon-2015 2.70%

The Activation-Induced Assembly of an RNA/Protein Interactome Centered on the Splicing Factor U2AF2 Regulates Gene Expression in Human CD4 T Cells. Whisenant et al (2015). *PLoS One*

Physical Interactions with 237 interactions from BioGRID

Lu-Zhang-2013 2.43%

The HECT type ubiquitin ligase NEDL2 is degraded by anaphase-promoting complex/cyclosome (APC/C)-Cdh1, and its tight regulation maintains the metaphase to anaphase transition. Lu et al (2013). *J Biol Chem*

Physical Interactions with 281 interactions from iRefIndex

Phillips-Corn-2013 1.88%

Conformational dynamics control ubiquitin-deubiquitinase interactions and influence in vivo signaling. Phillips et al (2013). *Proc Natl Acad Sci U S A*

Physical Interactions with 134 interactions from BioGRID

Wagner-Choudhary-2011 1.86%

A proteome-wide, quantitative survey of in vivo ubiquitylation sites reveals widespread regulatory roles. Wagner et al (2011). *Mol Cell Proteomics*

Physical Interactions with 1,158 interactions from iRefIndex

Behzadnia-Lührmann-2007 1.55%

Composition and three-dimensional EM structure of double affinity-purified, human prespliceosomal A complexes. Behzadnia et al (2007). *EMBO J*

Physical Interactions with 112 interactions from iRefIndex

Leung-Jones-2014 1.43%

Enhanced prediction of Src homology 2 (SH2) domain binding potentials using a fluorescence polarization-derived c-Met, c-Kit, ErbB, and androgen receptor interactome. Leung et al (2014). *Mol Cell Proteomics*

Physical Interactions with 190 interactions from iRefIndex

Maréchal-Zou-2014 1.41%

PRP19 transforms into a sensor of RPA-ssDNA after DNA damage and drives ATR activation via a ubiquitin-mediated circuitry. Maréchal et al (2014). *Mol Cell*

Physical Interactions with 976 interactions from iRefIndex

Berggård-James-2006 1.40%

Physical Interactions 67.64%

Berggård-James-2006

140 mouse brain proteins identified by Ca2+-calmodulin affinity chromatography and tandem mass spectrometry. Berggård et al (2006). *J Proteome Res*

Physical Interactions with 152 interactions from iRefIndex

Neganova-Lako-2011 1.28%

An important role for CDK2 in G1 to S checkpoint activation and DNA damage response in human embryonic stem cells. Neganova et al (2011). *Stem Cells*

Physical Interactions with 393 interactions from iRefIndex

McCracken-Blencowe-2005 1.21%

Proteomic analysis of SRm160-containing complexes reveals a conserved association with cohesin. McCracken et al (2005). *J Biol Chem*

Physical Interactions with 184 interactions from iRefIndex

Mak-Moffat-2010 1.16%

A lentiviral functional proteomics approach identifies chromatin remodeling complexes important for the induction of pluripotency. Mak et al (2010). *Mol Cell Proteomics*

Physical Interactions with 110 interactions from BioGRID

Murakawa-Landthaler-2015 1.03%

RC3H1 post-transcriptionally regulates A20 mRNA and modulates the activity of the IKK/NF- B pathway. Murakawa et al (2015). *Nat Commun*

Physical Interactions with 155 interactions from BioGRID

Llères-Lamond-2010 1.00%

Direct interaction between hnRNP-M and CDC5L/PLRG1 proteins affects alternative splice site choice. Llères et al (2010). *EMBO Rep*

Physical Interactions with 848 interactions from BioGRID

Nicholson-Hupp-2014 0.99%

A systems wide mass spectrometric based linear motif screen to identify dominant in-vivo interacting proteins for the ubiquitin ligase MDM2. Nicholson et al (2014). *Cell Signal*

Physical Interactions with 382 interactions from iRefIndex

Jones-MacBeath-2006 0.93%

A quantitative protein interaction network for the ErbB receptors using protein microarrays. Jones et al (2006). *Nature*

Physical Interactions with 151 interactions from iRefIndex

McFarland-Nussbaum-2008 0.87%

Proteomics analysis identifies phosphorylation-dependent alpha-synuclein protein interactions. McFarland et al (2008). *Mol Cell Proteomics*

Physical Interactions with 157 interactions from iRefIndex

Guarani-Harper-2014 0.83%

TIMMDC1/C3orf1 functions as a membrane-embedded mitochondrial complex I assembly factor through association with the MCIA complex. Guarani et al (2014). *Mol Cell Biol*

Physical Interactions with 323 interactions from BioGRID

Hill-Livingston-2014 0.83%

Systematic screening reveals a role for BRCA1 in the response to transcription-associated DNA damage. Hill et al (2014). *Genes Dev*

Physical Interactions with 125 interactions from iRefIndex

Physical Interactions 67.64%

Bett-Hay-2013 0.79%

The P-body component USP52/PAN2 is a novel regulator of HIF1A mRNA stability. Bett et al (2013). *Biochem J*

Physical Interactions with 319 interactions from iRefIndex

Stes-Gevaert-2014 0.73%

A COFRADIC protocol to study protein ubiquitination. Stes et al (2014). *J Proteome Res*

Physical Interactions with 1,327 interactions from iRefIndex

Danielsen-Nielsen-2011 0.67%

Mass spectrometric analysis of lysine ubiquitylation reveals promiscuity at site level. Danielsen et al (2011). *Mol Cell Proteomics*

Physical Interactions with 2,479 interactions from iRefIndex

Jeronimo-Coulombe-2007 0.65%

Systematic analysis of the protein interaction network for the human transcription machinery reveals the identity of the 7SK capping enzyme. Jeronimo et al (2007). *Mol Cell*

Physical Interactions with 699 interactions from BioGRID

Xu-Ye-2012 0.65%

SGTA recognizes a noncanonical ubiquitin-like domain in the Bag6-Ubl4A-Trc35 complex to promote endoplasmic reticulum-associated degradation. Xu et al (2012). *Cell Rep*

Physical Interactions with 225 interactions from iRefIndex

Gloeckner-Ueffing-2007 0.62%

A novel tandem affinity purification strategy for the efficient isolation and characterisation of native protein complexes. Gloeckner et al (2007). *Proteomics*

Physical Interactions with 100 interactions from BioGRID

Fogeron-Lange-2013 0.61%

LGALS3BP regulates centriole biogenesis and centrosome hypertrophy in cancer cells. Fogeron et al (2013). *Nat Commun*

Physical Interactions with 1,492 interactions from BioGRID

Freibaum-Taylor-2010 0.60%

Global analysis of TDP-43 interacting proteins reveals strong association with RNA splicing and translation machinery. Freibaum et al (2010). *J Proteome Res*

Physical Interactions with 216 interactions from iRefIndex

Zhou-Conrads-2004 0.59%

"An investigation into the human serum ""interactome""." Zhou et al (2004). *Electrophoresis*

Physical Interactions with 158 interactions from iRefIndex

Brehme-Superti-Furga-2009 0.59%

Charting the molecular network of the drug target Bcr-Abl. Brehme et al (2009). *Proc Natl Acad Sci U S A*

Physical Interactions with 578 interactions from iRefIndex

Yu-Chow-2013 0.59%

VCP phosphorylation-dependent interaction partners prevent apoptosis in Helicobacter pylori-infected gastric epithelial cells. Yu et al (2013). *PLoS One*

Physical Interactions with 272 interactions from iRefIndex

Hegele-Stelzl-2012 B 0.55%

Dynamic protein-protein interaction wiring of the human spliceosome. Hegele et al (2012). *Mol Cell*

Physical Interactions with 600 interactions from BioGRID

Physical Interactions 67.64%

Weinmann-Meister-2009 0.54%

Importin 8 is a gene silencing factor that targets argonaute proteins to distinct mRNAs. Weinmann et al (2009). *Cell*

Physical Interactions with 96 interactions from BioGRID

Narayan-Bennett-2012 0.53%

Short-chain 3-hydroxyacyl-coenzyme A dehydrogenase associates with a protein super-complex integrating multiple metabolic pathways. Narayan et al (2012). *PLoS One*

Physical Interactions with 110 interactions from BioGRID

Udeshi-Carr-2012 0.52%

Methods for quantification of in vivo changes in protein ubiquitination following proteasome and deubiquitinase inhibition. Udeshi et al (2012). *Mol Cell Proteomics*

Physical Interactions with 554 interactions from iRefIndex

Kristensen-Foster-2012 0.51%

A high-throughput approach for measuring temporal changes in the interactome. Kristensen et al (2012). *Nat Methods*

Physical Interactions with 7,115 interactions from BioGRID

Agrawal-Sedivy-2010 0.50%

Proteomic profiling of Myc-associated proteins. Agrawal et al (2010). *Cell Cycle*

Physical Interactions with 104 interactions from iRefIndex

Varjosalo-Superti-Furga-2013 0.49%

Interlaboratory reproducibility of large-scale human protein-complex analysis by standardized AP-MS. Varjosalo et al (2013). *Nat Methods*

Physical Interactions with 483 interactions from BioGRID

Rowbotham-Mermoud-2011 0.46%

Maintenance of silent chromatin through replication requires SWI/SNF-like chromatin remodeler SMARCAD1. Rowbotham et al (2011). *Mol Cell*

Physical Interactions with 114 interactions from iRefIndex

Arroyo-Aloy-2014 0.44%

Charting the molecular links between driver and susceptibility genes in colorectal cancer. Arroyo et al (2014). *Biochem Biophys Res Commun*

Physical Interactions with 598 interactions from iRefIndex

Barr-Knapp-2009 0.43%

Large-scale structural analysis of the classical human protein tyrosine phosphatome. Barr et al (2009). *Cell*

Physical Interactions with 164 interactions from iRefIndex

Emdal-Olsen-2015 0.41%

Temporal proteomics of NGF-TrkA signaling identifies an inhibitory role for the E3 ligase Cbl-b in neuroblastoma cell differentiation. Emdal et al (2015). *Sci Signal*

Physical Interactions with 1,919 interactions from BioGRID

Cox-Rizzino-2013 0.40%

The SOX2-interactome in brain cancer cells identifies the requirement of MSI2 and USP9X for the growth of brain tumor cells. Cox et al (2013). *PLoS One*

Physical Interactions with 280 interactions from iRefIndex

Giannone-Liu-2010 0.39%

Physical Interactions 67.64%

Giannone-Liu-2010

The protein network surrounding the human telomere repeat binding factors TRF1, TRF2, and POT1. Giannone et al (2010). *PLoS One*

Physical Interactions with 279 interactions from iRefIndex

Havugimana-Emili-2012 0.39%

A census of human soluble protein complexes. Havugimana et al (2012). *Cell*

Physical Interactions with 13,716 interactions from BioGRID

IREF-DIP 0.39%

Physical Interactions with 4,470 interactions from iRefIndex

Li-Dorf-2011 A 0.39%

Mapping a dynamic innate immunity protein interaction network regulating type I interferon production. Li et al (2011). *Immunity*

Physical Interactions with 400 interactions from BioGRID

Persaud-Rotin-2009 0.38%

Comparison of substrate specificity of the ubiquitin ligases Nedd4 and Nedd4-2 using proteome arrays. Persaud et al (2009). *Mol Syst Biol*

Physical Interactions with 239 interactions from iRefIndex

Foerster-Ritter-2013 0.37%

Characterization of the EGFR interactome reveals associated protein complex networks and intracellular receptor dynamics. Foerster et al (2013). *Proteomics*

Physical Interactions with 159 interactions from iRefIndex

Zhao-Krug-2005 0.36%

Human ISG15 conjugation targets both IFN-induced and constitutively expressed proteins functioning in diverse cellular pathways. Zhao et al (2005). *Proc Natl Acad Sci U S A*

Physical Interactions with 150 interactions from iRefIndex

Wan-Emili-2015 0.36%

Panorama of ancient metazoan macromolecular complexes. Wan et al (2015). *Nature*

Physical Interactions with 16,682 interactions from BioGRID

Fenner-Prehn-2010 0.36%

Expanding the substantial interactome of NEMO using protein microarrays. Fenner et al (2010). *PLoS One*

Physical Interactions with 103 interactions from iRefIndex

Roux-Burke-2012 0.35%

A promiscuous biotin ligase fusion protein identifies proximal and interacting proteins in mammalian cells. Roux et al (2012). *J Cell Biol*

Physical Interactions with 115 interactions from iRefIndex

Loch-Strickler-2012 0.33%

A microarray of ubiquitylated proteins for profiling deubiquitylase activity reveals the critical roles of both chain and substrate. Loch et al (2012). *Biochim Biophys Acta*

Physical Interactions with 145 interactions from iRefIndex

IREF-MATRIXDB 0.32%

Physical Interactions with 249 interactions from iRefIndex

Physical Interactions 67.64%

Diner-Cristea-2015 0.32%

Interactions of the Antiviral Factor Interferon Gamma-Inducible Protein 16 (IFI16) Mediate Immune Signaling and Herpes Simplex Virus-1 Immunosuppression. Diner et al (2015). *Mol Cell Proteomics*

Physical Interactions with 332 interactions from BioGRID

Hein-Mann-2015 0.31%

A human interactome in three quantitative dimensions organized by stoichiometries and abundances. Hein et al (2015). *Cell*

Physical Interactions with 27,044 interactions from BioGRID

Lipp-Guthrie-2015 0.30%

SR protein kinases promote splicing of nonconsensus introns. Lipp et al (2015). *Nat Struct Mol Biol*

Physical Interactions with 386 interactions from BioGRID

Li-Chen-2015 0.30%

Proteomic analyses reveal distinct chromatin-associated and soluble transcription factor complexes. Li et al (2015). *Mol Syst Biol*

Physical Interactions with 1,809 interactions from BioGRID

Wang-Xu-2015 0.29%

Interaction of amyotrophic lateral sclerosis/frontotemporal lobar degeneration-associated fused-in-sarcoma with proteins involved in metabolic and protein degradation pathways. Wang et al (2015). *Neurobiol Aging*

Physical Interactions with 192 interactions from iRefIndex

Roy-Pardo-2014 0.29%

hnRNPA1 couples nuclear export and translation of specific mRNAs downstream of FGF-2/S6K2 signalling. Roy et al (2014). *Nucleic Acids Res*

Physical Interactions with 386 interactions from BioGRID

Kotlyar-Jurisica-2015 0.28%

In silico prediction of physical protein interactions and characterization of interactome orphans. Kotlyar et al (2015). *Nat Methods*

Physical Interactions with 121 interactions from BioGRID

Barrios-Rodiles-Wrana-2005 0.27%

High-throughput mapping of a dynamic signaling network in mammalian cells. Barrios-Rodiles et al (2005). *Science*

Physical Interactions with 552 interactions from iRefIndex

BIOGRID-SMALL-SCALE-STUDIES 0.27%

Physical Interactions with 58,871 interactions from BioGRID

Sowa-Harper-2009 0.26%

Defining the human deubiquitinating enzyme interaction landscape. Sowa et al (2009). *Cell*

Physical Interactions with 1,509 interactions from BioGRID

Ramachandran-LaBaer-2004 0.26%

Self-assembling protein microarrays. Ramachandran et al (2004). *Science*

Physical Interactions with 112 interactions from iRefIndex

Oshikawa-Nakayama-2012 0.26%

Proteome-wide identification of ubiquitylation sites by conjugation of engineered lysine-less ubiquitin. Oshikawa et al (2012). *J Proteome Res*

Physical Interactions with 116 interactions from iRefIndex

Bonacci-Soubeyran-2014 0.26%

Physical Interactions 67.64%

Bonacci-Soubeyran-2014

Identification of new mechanisms of cellular response to chemotherapy by tracking changes in post-translational modifications by ubiquitin and ubiquitin-like proteins. Bonacci et al (2014). *J Proteome Res*

Physical Interactions with 937 interactions from iRefIndex

Kim-Gygi-2011 0.26%

Systematic and quantitative assessment of the ubiquitin-modified proteome. Kim et al (2011). *Mol Cell*

Physical Interactions with 1,345 interactions from iRefIndex

Taipale-Lindquist-2012 0.26%

Quantitative analysis of HSP90-client interactions reveals principles of substrate recognition. Taipale et al (2012). *Cell*

Physical Interactions with 716 interactions from iRefIndex

Wilker-Yaffe-2007 0.25%

14-3-3sigma controls mitotic translation to facilitate cytokinesis. Wilker et al (2007). *Nature*

Physical Interactions with 110 interactions from iRefIndex

Jin-Pawson-2004 0.24%

Proteomic, functional, and domain-based analysis of in vivo 14-3-3 binding proteins involved in cytoskeletal regulation and cellular organization. Jin et al (2004). *Curr Biol*

Physical Interactions with 236 interactions from iRefIndex

Oliviero-Cagney-2015 0.24%

The variant Polycomb Repressor Complex 1 component PCGF1 interacts with a pluripotency sub-network that includes DPPA4, a regulator of embryogenesis. Oliviero et al (2015). *Sci Rep*

Physical Interactions with 675 interactions from BioGRID

IREF-BIND 0.24%

Physical Interactions with 3,659 interactions from iRefIndex

Hutchins-Peters-2010 0.24%

Systematic analysis of human protein complexes identifies chromosome segregation proteins. Hutchins et al (2010). *Science*

Physical Interactions with 1,783 interactions from BioGRID

Tong-Moran-2014 0.23%

Proteomic analysis of the epidermal growth factor receptor (EGFR) interactome and post-translational modifications associated with receptor endocytosis in response to EGF and stress. Tong et al (2014). *Mol Cell Proteomics*

Physical Interactions with 271 interactions from iRefIndex

IREF-MPPI 0.23%

Physical Interactions with 382 interactions from iRefIndex

Li-Haura-2013 0.21%

Perturbation of the mutated EGFR interactome identifies vulnerabilities and resistance mechanisms. Li et al (2013). *Mol Syst Biol*

Physical Interactions with 403 interactions from BioGRID

Woods-Monteiro-2012 0.21%

Charting the landscape of tandem BRCT domain-mediated protein interactions. Woods et al (2012). *Sci Signal*

Physical Interactions with 919 interactions from iRefIndex

Xie-Cong-2013 0.19%

Physical Interactions 67.64%

Xie-Cong-2013

Deubiquitinase FAM/USP9X interacts with the E3 ubiquitin ligase SMURF1 protein and protects it from ligase activity-dependent self-degradation. Xie et al (2013). *J Biol Chem*

Physical Interactions with 168 interactions from iRefIndex

Malovannaya-Qin-2010 0.19%

Streamlined analysis schema for high-throughput identification of endogenous protein complexes. Malovannaya et al (2010). *Proc Natl Acad Sci U S A*

Physical Interactions with 224 interactions from iRefIndex

Chen-Zhang-2013 0.18%

Quantitative study of the interactome of PKC involved in the EGF-induced tumor cell chemotaxis. Chen et al (2013). *J Proteome Res*

Physical Interactions with 180 interactions from iRefIndex

Koch-Hermeking-2007 0.17%

Large-scale identification of c-MYC-associated proteins using a combined TAP/MudPIT approach. Koch et al (2007). *Cell Cycle*

Physical Interactions with 175 interactions from iRefIndex

Xiao-Lefkowitz-2007 0.17%

Functional specialization of beta-arrestin interactions revealed by proteomic analysis. Xiao et al (2007). *Proc Natl Acad Sci U S A*

Physical Interactions with 402 interactions from iRefIndex

Napolitano-Meroni-2011 0.17%

Functional interactions between ubiquitin E2 enzymes and TRIM proteins. Napolitano et al (2011). *Biochem J*

Physical Interactions with 81 interactions from BioGRID

IREF-PUBMED 0.16%

Physical Interactions with 571 interactions from iRefIndex

Varjosalo-Gstaiger-2013 0.16%

The protein interaction landscape of the human CMGC kinase group. Varjosalo et al (2013). *Cell Rep*

Physical Interactions with 936 interactions from iRefIndex

So-Colwill-2015 0.15%

Integrative analysis of kinase networks in TRAIL-induced apoptosis provides a source of potential targets for combination therapy. So et al (2015). *Sci Signal*

Physical Interactions with 647 interactions from BioGRID

Scholz-Taylor-2016 0.14%

FIH Regulates Cellular Metabolism through Hydroxylation of the Deubiquitinase OTUB1. Scholz et al (2016). *PLoS Biol*

Physical Interactions with 134 interactions from BioGRID

Huttlin-Gygi-2015 0.14%

The BioPlex Network: A Systematic Exploration of the Human Interactome. Huttlin et al (2015). *Cell*

Physical Interactions with 23,399 interactions from BioGRID

Ingham-Pawson-2005 0.14%

WW domains provide a platform for the assembly of multiprotein networks. Ingham et al (2005). *Mol Cell Biol*

Physical Interactions with 299 interactions from iRefIndex

Physical Interactions 67.64%

Tsai-Cristea-2012 0.13%

Functional proteomics establishes the interaction of SIRT7 with chromatin remodeling complexes and expands its role in regulation of RNA polymerase I transcription. Tsai et al (2012). *Mol Cell Proteomics*

Physical Interactions with 655 interactions from iRefIndex

Yatim-Benkirane-2012 0.12%

NOTCH1 nuclear interactome reveals key regulators of its transcriptional activity and oncogenic function. Yatim et al (2012). *Mol Cell*

Physical Interactions with 131 interactions from iRefIndex

Thompson-Luchansky-2014 0.12%

Quantitative Lys- -Gly-Gly (diGly) proteomics coupled with inducible RNAi reveals ubiquitin-mediated proteolysis of DNA damage-inducible transcript 4 (DDIT4) by the E3 ligase HUWE1. Thompson et al (2014). *J Biol Chem*

Physical Interactions with 552 interactions from iRefIndex

Floyd-Pagliarini-2016 0.12%

Mitochondrial Protein Interaction Mapping Identifies Regulators of Respiratory Chain Function. Floyd et al (2016). *Mol Cell*

Physical Interactions with 1,508 interactions from BioGRID

Bandyopadhyay-Ideker-2010 0.12%

A human MAP kinase interactome. Bandyopadhyay et al (2010). *Nat Methods*

Physical Interactions with 611 interactions from iRefIndex

van Wijk-Timmers-2009 0.11%

A comprehensive framework of E2-RING E3 interactions of the human ubiquitin-proteasome system. van Wijk et al (2009). *Mol Syst Biol*

Physical Interactions with 301 interactions from iRefIndex

IREF-INTACT 0.11%

Physical Interactions with 56,297 interactions from iRefIndex

Lee-Songyang-2011 0.11%

Genome-wide YFP fluorescence complementation screen identifies new regulators for telomere signaling in human cells. Lee et al (2011). *Mol Cell Proteomics*

Physical Interactions with 604 interactions from iRefIndex

Grossmann-Stelzl-2015 0.11%

Phospho-tyrosine dependent protein-protein interaction network. Grossmann et al (2015). *Mol Syst Biol*

Physical Interactions with 622 interactions from BioGRID

Liu-Wang-2012 0.11%

Proteomic identification of common SCF ubiquitin ligase FBXO6-interacting glycoproteins in three kinds of cells. Liu et al (2012). *J Proteome Res*

Physical Interactions with 586 interactions from iRefIndex

Povlsen-Choudhary-2012 0.10%

Systems-wide analysis of ubiquitylation dynamics reveals a key role for PAF15 ubiquitylation in DNA-damage bypass. Povlsen et al (2012). *Nat Cell Biol*

Physical Interactions with 562 interactions from iRefIndex

Oláh-Ovádi-2011 0.10%

Interactions of pathological hallmark proteins: tubulin polymerization promoting protein/p25, beta-amyloid, and alpha-synuclein. Oláh et al (2011). *J Biol Chem*

Physical Interactions 67.64%

Oláh-Ovádi-2011

Physical Interactions with 1,853 interactions from iRefIndex

Hayes-Urbé-2012 0.10%

Direct and indirect control of mitogen-activated protein kinase pathway-associated components, BRAP/IMP E3 ubiquitin ligase and CRAF/RAF1 kinase, by the deubiquitylating enzyme USP15. Hayes et al (2012). *J Biol Chem*

Physical Interactions with 107 interactions from iRefIndex

Joshi-Cristea-2013 0.10%

The functional interactome landscape of the human histone deacetylase family. Joshi et al (2013). *Mol Syst Biol*

Physical Interactions with 310 interactions from iRefIndex

Roy-Parent-2013 0.10%

Novel, gel-free proteomics approach identifies RNF5 and JAMP as modulators of GPCR stability. Roy et al (2013). *Mol Endocrinol*

Physical Interactions with 155 interactions from iRefIndex

Ewing-Figeys-2007 0.10%

Large-scale mapping of human protein-protein interactions by mass spectrometry. Ewing et al (2007). *Mol Syst Biol*

Physical Interactions with 5,362 interactions from iRefIndex

Zanon-Pichler-2013 0.09%

Profiling of Parkin-binding partners using tandem affinity purification. Zanon et al (2013). *PLoS One*

Physical Interactions with 187 interactions from iRefIndex

Brajenovic-Drewes-2004 0.09%

Comprehensive proteomic analysis of human Par protein complexes reveals an interconnected protein network. Brajenovic et al (2004). *J Biol Chem*

Physical Interactions with 141 interactions from iRefIndex

Greco-Cristea-2011 0.09%

Nuclear import of histone deacetylase 5 by requisite nuclear localization signal phosphorylation. Greco et al (2011). *Mol Cell Proteomics*

Physical Interactions with 240 interactions from iRefIndex

Wu-Li-2007 0.09%

Systematic identification of SH3 domain-mediated human protein-protein interactions by peptide array target screening. Wu et al (2007). *Proteomics*

Physical Interactions with 927 interactions from iRefIndex

Behrends-Harper-2010 0.09%

Network organization of the human autophagy system. Behrends et al (2010). *Nature*

Physical Interactions with 751 interactions from iRefIndex

Nakayasu-Adkins-2013 0.09%

Evaluation of selected binding domains for the analysis of ubiquitinated proteomes. Nakayasu et al (2013). *J Am Soc Mass Spectrom*

Physical Interactions with 880 interactions from iRefIndex

Ouyang-Gill-2009 0.08%

Direct binding of CoREST1 to SUMO-2/3 contributes to gene-specific repression by the LSD1/CoREST1/HDAC complex. Ouyang et al (2009). *Mol Cell*

Physical Interactions 67.64%

Ouyang-Gill-2009

Physical Interactions with 105 interactions from BioGRID

Perez-Hernandez-Yáñez-Mó-2013 0.08%

The intracellular interactome of tetraspanin-enriched microdomains reveals their function as sorting machineries toward exosomes. Perez-Hernandez et al (2013). *J Biol Chem*

Physical Interactions with 450 interactions from iRefIndex

Reyniers-Taymans-2014 0.08%

Differential protein-protein interactions of LRRK1 and LRRK2 indicate roles in distinct cellular signaling pathways. Reyniers et al (2014). *J Neurochem*

Physical Interactions with 102 interactions from iRefIndex

Bruderer-Hay-2011 0.08%

Purification and identification of endogenous polySUMO conjugates. Bruderer et al (2011). *EMBO Rep*

Physical Interactions with 106 interactions from iRefIndex

Couzens-Gingras-2013 0.08%

Protein interaction network of the mammalian Hippo pathway reveals mechanisms of kinase-phosphatase interactions. Couzens et al (2013). *Sci Signal*

Physical Interactions with 364 interactions from BioGRID

Bennett-Harper-2010 0.08%

Dynamics of cullin-RING ubiquitin ligase network revealed by systematic quantitative proteomics. Bennett et al (2010). *Cell*

Physical Interactions with 4,367 interactions from BioGRID

Christianson-Kopito-2011 0.07%

Defining human ERAD networks through an integrative mapping strategy. Christianson et al (2011). *Nat Cell Biol*

Physical Interactions with 260 interactions from iRefIndex

Albers-Koegl-2005 0.07%

Automated yeast two-hybrid screening for nuclear receptor-interacting proteins. Albers et al (2005). *Mol Cell Proteomics*

Physical Interactions with 238 interactions from iRefIndex

Thalappilly-Dusetti-2008 0.07%

Identification of multi-SH3 domain-containing protein interactome in pancreatic cancer: a yeast two-hybrid approach. Thalappilly et al (2008). *Proteomics*

Physical Interactions with 104 interactions from iRefIndex

Gupta-Pelletier-2015 0.07%

A Dynamic Protein Interaction Landscape of the Human Centrosome-Cilium Interface. Gupta et al (2015). *Cell*

Physical Interactions with 307 interactions from BioGRID

Vinayagam-Wanker-2011 0.06%

A directed protein interaction network for investigating intracellular signal transduction. Vinayagam et al (2011). *Sci Signal*

Physical Interactions with 2,576 interactions from BioGRID

Li-Dorf-2014 0.06%

TRIM65 regulates microRNA activity by ubiquitination of TNRC6. Li et al (2014). *Proc Natl Acad Sci U S A*

Physical Interactions with 470 interactions from iRefIndex

Katsogiannou-Rocchi-2014 0.06%

Physical Interactions 67.64%

Katsogiannou-Rocchi-2014

The functional landscape of Hsp27 reveals new cellular processes such as DNA repair and alternative splicing and proposes novel anticancer targets. Katsogiannou et al (2014). *Mol Cell Proteomics*

Physical Interactions with 217 interactions from iRefIndex

Arbuckle-Grant-2010 0.05%

The SH3 domain of postsynaptic density 95 mediates inflammatory pain through phosphatidylinositol-3-kinase recruitment. Arbuckle et al (2010). *EMBO Rep*

Physical Interactions with 268 interactions from iRefIndex

Bantscheff-Drewes-2011 0.05%

Chemoproteomics profiling of HDAC inhibitors reveals selective targeting of HDAC complexes. Bantscheff et al (2011). *Nat Biotechnol*

Physical Interactions with 103 interactions from BioGRID

Low-Heck-2014 0.04%

A systems-wide screen identifies substrates of the SCF TrCP ubiquitin ligase. Low et al (2014). *Sci Signal*

Physical Interactions with 221 interactions from BioGRID

Lau-Ronai-2012 0.04%

PKC promotes oncogenic functions of ATF2 in the nucleus while blocking its apoptotic function at mitochondria. Lau et al (2012). *Cell*

Physical Interactions with 134 interactions from iRefIndex

Kırlı-Görlich-2015 0.04%

A deep proteomics perspective on CRM1-mediated nuclear export and nucleocytoplasmic partitioning. Kırlı et al (2015). *Elife*

Physical Interactions with 1,036 interactions from BioGRID

Woodsmith-Sanderson-2012 0.04%

Systematic analysis of dimeric E3-RING interactions reveals increased combinatorial complexity in human ubiquitination networks. Woodsmith et al (2012). *Mol Cell Proteomics*

Physical Interactions with 212 interactions from iRefIndex

Zhang-Zou-2011 0.03%

A bead-based approach for large-scale identification of in vitro kinase substrates. Zhang et al (2011). *Proteomics*

Physical Interactions with 162 interactions from iRefIndex

Leng-Wang-2014 0.03%

A proteomics strategy for the identification of FAT10-modified sites by mass spectrometry. Leng et al (2014). *J Proteome Res*

Physical Interactions with 144 interactions from iRefIndex

Bogachek-Weigel-2014 0.02%

Sumoylation pathway is required to maintain the basal breast cancer subtype. Bogachek et al (2014). *Cancer Cell*

Physical Interactions with 134 interactions from iRefIndex

Hanson-Clayton-2014 0.02%

Identifying biological pathways that underlie primordial short stature using network analysis. Hanson et al (2014). *J Mol Endocrinol*

Physical Interactions with 1,687 interactions from iRefIndex

Markson-Sanderson-2009 0.02%

Analysis of the human E2 ubiquitin conjugating enzyme protein interaction network. Markson et al (2009). *Genome Res*

Physical Interactions 67.64%

Markson-Sanderson-2009

Physical Interactions with 700 interactions from iRefIndex

Golebiowski-Hay-2009 0.02%

System-wide changes to SUMO modifications in response to heat shock. Golebiowski et al (2009). *Sci Signal*

Physical Interactions with 351 interactions from iRefIndex

Yang-Chen-2010 0.02%

Proteomic dissection of cell type-specific H2AX-interacting protein complex associated with hepatocellular carcinoma. Yang et al (2010). *J Proteome Res*

Physical Interactions with 100 interactions from BioGRID

Ravasi-Hayashizaki-2010 0.02%

An atlas of combinatorial transcriptional regulation in mouse and man. Ravasi et al (2010). *Cell*

Physical Interactions with 635 interactions from iRefIndex

Arroyo-Aloy-2015 0.02%

Systematic identification of molecular links between core and candidate genes in breast cancer. Arroyo et al (2015). *J Mol Biol*

Physical Interactions with 600 interactions from iRefIndex

IREF-HPRD 0.02%

Physical Interactions with 34,206 interactions from iRefIndex

Pichlmair-Superti-Furga-2012 0.02%

Viral immune modulators perturb the human molecular network by common and unique strategies. Pichlmair et al (2012). *Nature*

Physical Interactions with 14 interactions from BioGRID

Virok-Fülöp-2011 0.02%

Protein array based interactome analysis of amyloid- indicates an inhibition of protein translation. Virok et al (2011). *J Proteome Res*

Physical Interactions with 299 interactions from BioGRID

Vermeulen-Mann-2010 0.01%

Quantitative interaction proteomics and genome-wide profiling of epigenetic histone marks and their readers. Vermeulen et al (2010). *Cell*

Physical Interactions with 131 interactions from iRefIndex

Grant-2010 0.01%

Identification of SUMOylated proteins in neuroblastoma cells after treatment with hydrogen peroxide or ascorbate. Grant (2010). *BMB Rep*

Physical Interactions with 114 interactions from iRefIndex

Meek-Piwnica-Worms-2004 0.01%

Comprehensive proteomic analysis of interphase and mitotic 14-3-3-binding proteins. Meek et al (2004). *J Biol Chem*

Physical Interactions with 359 interactions from iRefIndex

Soler-López-Aloy-2011 0.01%

Interactome mapping suggests new mechanistic details underlying Alzheimer's disease. Soler-López et al (2011). *Genome Res*

Physical Interactions with 312 interactions from iRefIndex

Yu-Vidal-2011 0.01%

Next-generation sequencing to generate interactome datasets. Yu et al (2011). *Nat Methods*

Physical Interactions 67.64%

Yu-Vidal-2011

Physical Interactions with 1,108 interactions from BioGRID

Weimann-Stelzl-2013 A 0.00%

A Y2H-seq approach defines the human protein methyltransferase interactome. Weimann et al (2013). *Nat Methods*

Physical Interactions with 114 interactions from BioGRID

Wong-O'Bryan-2012 0.00%

Intersectin (ITSN) family of scaffolds function as molecular hubs in protein interaction networks. Wong et al (2012). *PLoS One*

Physical Interactions with 111 interactions from iRefIndex

Kim-Major-2015 0.00%

Substrate trapping proteomics reveals targets of the TrCP2/FBXW11 ubiquitin ligase. Kim et al (2015). *Mol Cell Biol*

Physical Interactions with 114 interactions from iRefIndex

Co-expression 13.50%

Ramaswamy-Golub-2001 1.02%

Multiclass cancer diagnosis using tumor gene expression signatures. Ramaswamy et al (2001). *Proc Natl Acad Sci U S A*

Co-expression with 275,113 interactions from supplementary material

Wang-Maris-2006 0.95%

Integrative genomics identifies distinct molecular classes of neuroblastoma and shows that multiple genes are targeted by regional alterations in DNA copy number. Wang et al (2006). *Cancer Res*

Co-expression with 264,023 interactions from GEO

Mallon-McKay-2013 0.86%

StemCellDB: the human pluripotent stem cell database at the National Institutes of Health. Mallon et al (2013). *Stem Cell Res*

Co-expression with 585,265 interactions from GEO

Bild-Nevins-2006 B 0.85%

Oncogenic pathway signatures in human cancers as a guide to targeted therapies. Bild et al (2006). *Nature*

Co-expression with 280,683 interactions from GEO

Burington-Shaughnessy-2008 0.80%

Tumor cell gene expression changes following short-term in vivo exposure to single agent chemotherapeutics are related to survival in multiple myeloma. Burington et al (2008). *Clin Cancer Res*

Co-expression with 290,538 interactions from GEO

Dobbin-Giordano-2005 0.78%

Interlaboratory comparability study of cancer gene expression analysis using oligonucleotide microarrays. Dobbin et al (2005). *Clin Cancer Res*

Co-expression with 444,931 interactions from GEO

Bahr-Bowler-2013 0.70%

Peripheral blood mononuclear cell gene expression in chronic obstructive pulmonary disease. Bahr et al (2013). *Am J Respir Cell Mol Biol*

Co-expression with 274,949 interactions from GEO

Alizadeh-Staudt-2000 0.69%

Distinct types of diffuse large B-cell lymphoma identified by gene expression profiling. Alizadeh et al (2000). *Nature*

Co-expression with 90,336 interactions from supplementary material

Co-expression 13.50%

Innocenti-Brown-2011 0.69%

Identification, replication, and functional fine-mapping of expression quantitative trait loci in primary human liver tissue. Innocenti et al (2011). *PLoS Genet*

Co-expression with 603,765 interactions from GEO

Rieger-Chu-2004 0.66%

Toxicity from radiation therapy associated with abnormal transcriptional responses to DNA damage. Rieger et al (2004). *Proc Natl Acad Sci U S A*

Co-expression with 259,974 interactions from GEO

Noble-Diehl-2008 0.65%

Regional variation in gene expression in the healthy colon is dysregulated in ulcerative colitis. Noble et al (2008). *Gut*

Co-expression with 661,539 interactions from GEO

Roth-Zlotnik-2006 0.61%

Gene expression analyses reveal molecular relationships among 20 regions of the human CNS. Roth et al (2006). *Neurogenetics*

Co-expression with 669,062 interactions from GEO

Boldrick-Relman-2002 0.60%

Stereotyped and specific gene expression programs in human innate immune responses to bacteria. Boldrick et al (2002). *Proc Natl Acad Sci U S A*

Co-expression with 111,707 interactions from supplementary material

Perou-Botstein-2000 0.59%

Molecular portraits of human breast tumours. Perou et al (2000). *Nature*

Co-expression with 185,068 interactions from supplementary material

Smirnov-Cheung-2009 0.58%

Genetic analysis of radiation-induced changes in human gene expression. Smirnov et al (2009). *Nature*

Co-expression with 461,500 interactions from GEO

Wang-Cheung-2015 0.57%

Genetic variation in insulin-induced kinase signaling. Wang et al (2015). *Mol Syst Biol*

Co-expression with 411,047 interactions from GEO

Chen-Brown-2002 0.51%

Gene expression patterns in human liver cancers. Chen et al (2002). *Mol Biol Cell*

Co-expression with 282,241 interactions from supplementary material

Perou-Botstein-1999 0.50%

Distinctive gene expression patterns in human mammary epithelial cells and breast cancers. Perou et al (1999). *Proc Natl Acad Sci U S A*

Co-expression with 65,069 interactions from supplementary material

Wu-Garvey-2007 0.48%

The effect of insulin on expression of genes and biochemical pathways in human skeletal muscle. Wu et al (2007). *Endocrine*

Co-expression with 267,109 interactions from GEO

Rosenwald-Staudt-2001 0.40%

Relation of gene expression phenotype to immunoglobulin mutation genotype in B cell chronic lymphocytic leukemia. Rosenwald et al (2001). *J Exp Med*

Co-expression with 114,694 interactions from supplementary material

**Predicted**	6.35%

I2D-vonMering-Bork-2002-High-Yeast2Human — 0.89%

Comparative assessment of large-scale data sets of protein-protein interactions. von Mering et al (2002). *Nature*

Predicted with 1,196 interactions from I2D

I2D-vonMering-Bork-2002-Medium-Yeast2Human — 0.59%

Comparative assessment of large-scale data sets of protein-protein interactions. von Mering et al (2002). *Nature*

Predicted with 3,009 interactions from I2D

I2D-BioGRID-Yeast2Human — 0.50%

BioGRID: a general repository for interaction datasets. Stark et al (2006). *Nucleic Acids Res*

Predicted with 13,434 interactions from I2D

I2D-Chen-Pawson-2009-PiwiScreen-Mouse2Human — 0.46%

Mouse Piwi interactome identifies binding mechanism of Tdrkh Tudor domain to arginine methylated Miwi. Chen et al (2009). *Proc Natl Acad Sci U S A*

Predicted with 31 interactions from I2D

I2D-INNATEDB-Mouse2Human — 0.42%

InnateDB: facilitating systems-level analyses of the mammalian innate immune response. Lynn et al (2008). *Mol Syst Biol*

Predicted with 1,451 interactions from I2D

I2D-Tarassov-PCA-Yeast2Human — 0.40%

An in vivo map of the yeast protein interactome. Tarassov et al (2008). *Science*

Predicted with 440 interactions from I2D

I2D-vonMering-Bork-2002-Low-Yeast2Human — 0.38%

Comparative assessment of large-scale data sets of protein-protein interactions. von Mering et al (2002). *Nature*

Predicted with 16,063 interactions from I2D

Wu-Stein-2010 — 0.34%

A human functional protein interaction network and its application to cancer data analysis. Wu et al (2010). *Genome Biol*

Predicted with 87,829 interactions from supplementary material

I2D-IntAct-Mouse2Human — 0.31%

The IntAct molecular interaction database in 2010. Aranda et al (2010). *Nucleic Acids Res*

Predicted with 3,427 interactions from I2D

Stuart-Kim-2003 — 0.27%

A gene-coexpression network for global discovery of conserved genetic modules. Stuart et al (2003). *Science*

Predicted with 24,872 interactions from supplementary material

I2D-Yu-Vidal-2008-GoldStd-Yeast2Human — 0.22%

High-quality binary protein interaction map of the yeast interactome network. Yu et al (2008). *Science*

Predicted with 386 interactions from I2D

I2D-Krogan-Greenblatt-2006-Core-Yeast2Human — 0.19%

Global landscape of protein complexes in the yeast Saccharomyces cerevisiae. Krogan et al (2006). *Nature*

Predicted with 1,823 interactions from I2D

I2D-BIND-Rat2Human — 0.19%

BIND--a data specification for storing and describing biomolecular interactions, molecular complexes and pathways. Bader et al

Predicted 6.35%

I2D-BIND-Rat2Human

(2000). *Bioinformatics*

Predicted with 548 interactions from I2D

I2D-BioGRID-Worm2Human 0.18%

BioGRID: a general repository for interaction datasets. Stark et al (2006). *Nucleic Acids Res*

Predicted with 952 interactions from I2D

I2D-MINT-Rat2Human 0.17%

MINT: a Molecular INTeraction database. Zanzoni et al (2002). *FEBS Lett*

Predicted with 572 interactions from I2D

I2D-MINT-Mouse2Human 0.15%

MINT: a Molecular INTeraction database. Zanzoni et al (2002). *FEBS Lett*

Predicted with 971 interactions from I2D

I2D-BIND-Mouse2Human 0.14%

BIND--a data specification for storing and describing biomolecular interactions, molecular complexes and pathways. Bader et al (2000). *Bioinformatics*

Predicted with 1,186 interactions from I2D

I2D-BioGRID-Mouse2Human 0.12%

BioGRID: a general repository for interaction datasets. Stark et al (2006). *Nucleic Acids Res*

Predicted with 286 interactions from I2D

I2D-BIND-Yeast2Human 0.11%

BIND--a data specification for storing and describing biomolecular interactions, molecular complexes and pathways. Bader et al (2000). *Bioinformatics*

Predicted with 1,541 interactions from I2D

I2D-Krogan-Greenblatt-2006-NonCore-Yeast2Human 0.09%

Global landscape of protein complexes in the yeast Saccharomyces cerevisiae. Krogan et al (2006). *Nature*

Predicted with 1,786 interactions from I2D

I2D-IntAct-Fly2Human 0.09%

The IntAct molecular interaction database in 2010. Aranda et al (2010). *Nucleic Acids Res*

Predicted with 3,912 interactions from I2D

I2D-Formstecher-Daviet-2005-Embryo-Fly2Human 0.07%

Protein interaction mapping: a Drosophila case study. Formstecher et al (2005). *Genome Res*

Predicted with 491 interactions from I2D

I2D-MGI-Mouse2Human 0.07%

Ontological visualization of protein-protein interactions. Drabkin et al (2005). *BMC Bioinformatics*

Predicted with 726 interactions from I2D

I2D-IntAct-Rat2Human 0.01%

The IntAct molecular interaction database in 2010. Aranda et al (2010). *Nucleic Acids Res*

Predicted with 1,052 interactions from I2D

I2D-MINT-Worm2Human 0.00%

Predicted 6.35%

I2D-MINT-Worm2Human

MINT: a Molecular INTeraction database. Zanzoni et al (2002). *FEBS Lett*

Predicted with 1,178 interactions from I2D

Co-localization 6.17%

Zhang-Shang-2006 2.58%

The catalytic subunit of the proteasome is engaged in the entire process of estrogen receptor-regulated transcription. Zhang et al (2006). *EMBO J*

Co-localization with 53 interactions from BioGRID

Schadt-Shoemaker-2004 1.65%

A comprehensive transcript index of the human genome generated using microarrays and computational approaches. Schadt et al (2004). *Genome Biol*

Co-localization with 60,126 interactions from GEO

Johnson-Shoemaker-2003 1.11%

Genome-wide survey of human alternative pre-mRNA splicing with exon junction microarrays. Johnson et al (2003). *Science*

Co-localization with 426,332 interactions from GEO

Chen-Huang-2014 0.82%

Using an in situ proximity ligation assay to systematically profile endogenous protein-protein interactions in a pathway network. Chen et al (2014). *J Proteome Res*

Co-localization with 559 interactions from BioGRID

Pathway 4.35%

Wu-Stein-2010 1.54%

A human functional protein interaction network and its application to cancer data analysis. Wu et al (2010). *Genome Biol*

Pathway with 78,010 interactions from supplementary material

REACTOME 1.33%

Pathway with 24,913 interactions from Pathway Commons

NCI_NATURE 0.58%

Pathway with 10,122 interactions from Pathway Commons

CELL_MAP 0.46%

Pathway with 598 interactions from Pathway Commons

IMID 0.41%

Pathway with 1,073 interactions from Pathway Commons

HUMANCYC 0.03%

Pathway with 680 interactions from Pathway Commons

Genetic Interactions 1.40%

Vizeacoumar-Moffat-2013 0.41%

A negative genetic interaction map in isogenic cancer cell lines reveals cancer cell vulnerabilities. Vizeacoumar et al (2013). *Mol Syst Biol*

Genetic Interactions with 201 interactions from BioGRID

BIOGRID-SMALL-SCALE-STUDIES 0.38%

Genetic Interactions 1.40%

BIOGRID-SMALL-SCALE-STUDIES

Genetic Interactions with 489 interactions from BioGRID

Toyoshima-Grandori-2012 0.28%

Functional genomics identifies therapeutic targets for MYC-driven cancer. Toyoshima et al (2012). *Proc Natl Acad Sci U S A*

Genetic Interactions with 101 interactions from BioGRID

IREF-SMALL-SCALE-STUDIES 0.24%

Genetic Interactions with 35 interactions from iRefIndex

Blomen-Brummelkamp-2015 0.08%

Gene essentiality and synthetic lethality in haploid human cells. Blomen et al (2015). *Science*

Genetic Interactions with 127 interactions from BioGRID

Willingham-Muchowski-2003 0.00%

Yeast genes that enhance the toxicity of a mutant huntingtin fragment or alpha-synuclein. Willingham et al (2003). *Science*

Genetic Interactions with 37 interactions from BioGRID

Shared protein domains 0.59%

INTERPRO 0.38%

Shared protein domains with 608,863 interactions from InterPro

PFAM 0.21%

Shared protein domains with 457,054 interactions from Pfam
